# The Venus flytrap trigger hair–specific potassium channel KDM1 can reestablish the K^+^ gradient required for hapto-electric signaling

**DOI:** 10.1371/journal.pbio.3000964

**Published:** 2020-12-09

**Authors:** Anda L. Iosip, Jennifer Böhm, Sönke Scherzer, Khaled A. S. Al-Rasheid, Ingo Dreyer, Jörg Schultz, Dirk Becker, Ines Kreuzer, Rainer Hedrich

**Affiliations:** 1 Institute for Molecular Plant Physiology and Biophysics, University of Würzburg, Würzburg, Germany; 2 Center for Computational and Theoretical Biology, University of Würzburg, Würzburg, Germany; 3 Zoology Department, College of Science, King Saud University, Riyadh, Saudi Arabia; 4 Center of Bioinformatics, Simulation and Modeling (CBSM), Faculty of Engineering, Universidad de Talca, Talca, Chile; UCSD, UNITED STATES

## Abstract

The carnivorous plant *Dionaea muscipula* harbors multicellular trigger hairs designed to sense mechanical stimuli upon contact with animal prey. At the base of the trigger hair, mechanosensation is transduced into an all-or-nothing action potential (AP) that spreads all over the trap, ultimately leading to trap closure and prey capture. To reveal the molecular basis for the unique functional repertoire of this mechanoresponsive plant structure, we determined the transcriptome of *D*. *muscipula*’s trigger hair. Among the genes that were found to be highly specific to the trigger hair, the Shaker-type channel KDM1 was electrophysiologically characterized as a hyperpolarization- and acid-activated K^+^-selective channel, thus allowing the reuptake of K^+^ ions into the trigger hair’s sensory cells during the hyperpolarization phase of the AP. During trap development, the increased electrical excitability of the trigger hair is associated with the transcriptional induction of *KDM1*. Conversely, when KDM1 is blocked by Cs^+^ in adult traps, the initiation of APs in response to trigger hair deflection is reduced, and trap closure is suppressed. KDM1 thus plays a dominant role in K^+^ homeostasis in the context of AP and turgor formation underlying the mechanosensation of trigger hair cells and thus *D*. *muscipula*’s hapto-electric signaling.

## Abstract 2

Transcriptomic and electrophysiological studies of the carnivorous Venus flytrap reveal that potassium uptake via a trigger hair-specific potassium channel builds the basis for mechanosensation of likely prey and generation of an action potential that triggers closure of the trap.

## Introduction

Even though all plants are able to respond to mechanical forces, the trap closure mechanism of the carnivorous Venus flytrap (*Dionaea muscipula*) is one of the most spectacular and rapid thigmonastic movements in the plant kingdom [1]. The upper part of *D*. *muscipula’s* leaf has evolved into a bilobed snap trap equipped with touch-responsive trigger hairs that translate the mechanical stimulus from insects into electrical signals. A deflection of just 2.9° combined with a micronewton force suffices to elicit an action potential (AP) that originates at the base of the trigger hair and spreads along the entire trap surface [2]. The hair’s hypersensitivity to touch is provided by its unique biomechanical and biophysical properties, as well as its special anatomy and morphology. The hair comprises 2 distinct parts: The upper 1.5-mm slender cone, called the lever, is composed of elongated thick-walled terminal cells, whose main function is to amplify the mechanical stimulus, while the 0.15-mm basal podium anchors the hair into the underlying trap tissue [3,4]. Between the lever and the podium, there is a deep constriction site (indentation zone) where the bending of the trigger hair translates into shear stress. Here, a single-layered ring of as few as 50 highly specialized mechanoreceptor cells drive the mechanotransduction [3,5]. Both the outer and inner tangential cell walls of these sensory cells are thickened, but the transversal walls are comparatively thin and packed with numerous plasmodesmata at the basal side, which might serve to facilitate symplastic communication with the underlying podium cells. Structurally, the mechanoreceptor cells show an organized polarity, which is achieved through the localization of concentrically arranged endoplasmic reticula (ER) in both the apical and the basal pole. The central part of the cells, meanwhile, is occupied by numerous mitochondria, small tannin depositing vacuoles, lipid droplets, few plastids, very few ribosomes, and the nucleus [5,6]. The ER and the cell wall are both major Ca^2+^ storage sites, which is significant because, in *D*. *muscipula*, mechanical stimulation triggers both an electrical and a Ca^2+^ wave [2,7]. It is worth mentioning that an AP can also be elicited by compressing the trap’s non-trigger-hair tissue by a force more than 1,000 times greater than the one that is sufficient to evoke a trigger hair–dependent electrical response [2]. Although collision with an insect will not exert such a high force, it shows that cells other than those of the trigger hair are equipped with mechanosensors and inherently have the ability to initiate an AP. In that regard, it appears that the trigger hair functions as a lever that amplifies the force on the sensory cells, so that a prey as light as a 3-mg mosquito will elicit an AP in small *D*. *muscipula* traps. In order to catch the prey successfully, however, 2 APs need to be elicited within 30 seconds in order to pass the cytoplasmic Ca^2+^ threshold required to snap the trap shut [8,9]. The ongoing mechanical stimulation provided by the struggling prey then encourages the trap to close more tightly, until eventually it is hermetically sealed. In addition, more than 5 APs will initiate the expression of genes encoding hydrolases that make up a digestive cocktail [10], as well as transporters that will take up the released nutrients [11,12]. To unravel the molecular basis for mechanoperception and AP initiation in the trigger hair, we analyzed its transcriptomic landscape and identified the molecular elements giving rise to its unique hapto-electrical properties.

## Results

### Identification of trigger hair–specific genes

In order to characterize the transcriptome of the trigger hair, we collected trigger hairs from adult traps and sequenced their cDNA on an Illumina High Seq platform (San Diego, California, United States of America). For quantification, resulting reads, as well as those from 6 other organs (flower, root, petiole, whole trap, trap rim, and gland) [8], were mapped onto the draft genome of *D*. *muscipula* [13].

Integrating variance-stabilized expression data from all available tissues in the non-stimulated state, the principal component analysis (PCA; S1A Fig) showed that all 3 replicates of each tissue sample grouped together. The first 2 dimensions already accounted for 78% of the overall variance. The component with the largest spread (PC1, 59%) separated root and flower from the photosynthetically active petiole and trap, including specific trap tissues such as rim, gland, and trigger hair. The trigger hair replicates clearly separated from petiole and trap rim, thus clustering with the electrically excitable trap tissue. This pattern was confirmed by pairwise Pearson-type global correlation measurement (Pearson correlation value of 0.76 for trigger hair versus trap; S1B Fig).

**Fig 1:**
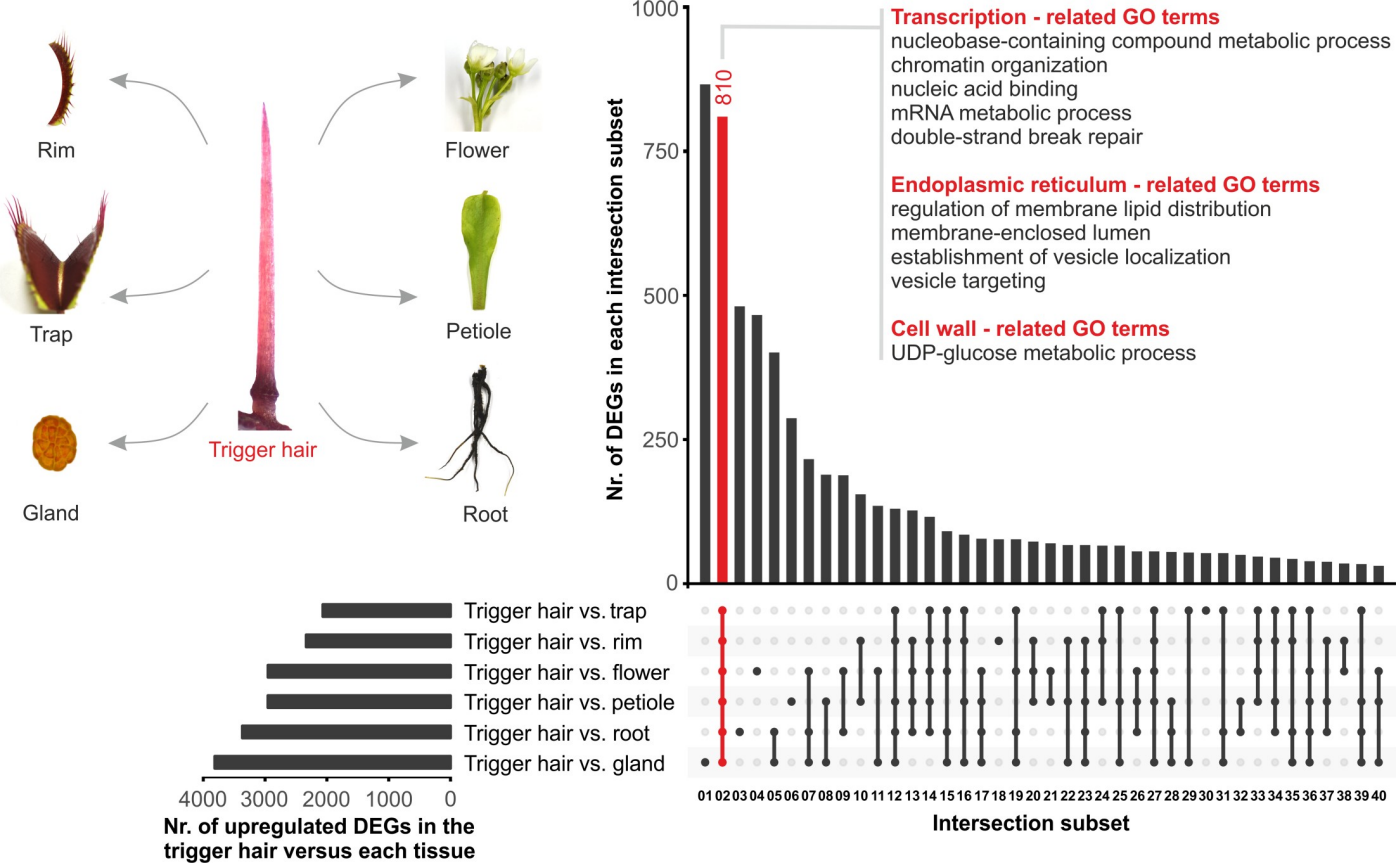
A high number of genes are highly expressed in the trigger hair. Of note, the expression of 810 DEGs is elevated by a factor of at least 2 in the trigger hairs compared to all of the other tissues such as trap, rim, flower, petiole, root, and gland tissues (log_2_FC > 1, BH adjusted *p*-value < 0.001, trigger hair normalized counts > 50). Enriched GO terms for subset02 (810 genes, red bar, representing trigger hair vs. all tissues) of the intersection analysis between DEGs groups are shown (GO enrichment, BH adjusted *p*-value < 0.05). Underlying full raw dataset is provided in S1 Data. BH, Benjamini–Hochberg; DEG, differentially expressed gene; GO, gene ontology; UDP-glucose, uridine diphosphate glucose.

To identify trigger hair–specific transcripts, we integrated 2 complementary methods (see Materials and methods section). First, we performed an intersection analysis of significantly (Benjamini–Hochberg (BH) adjusted *p*-value < 0.001) differentially expressed genes (DEGs) (Fig 1, S1 Data). Remarkably, a high number of DEGs (810) (Fig 1, subset 2 marked in red) were up-regulated by at least 2-fold in trigger hairs compared to all other tissues, supporting the idea that this special mechanosensory structure is characterized by a unique transcriptomic landscape. In order to identify the major functions hardwired in the trigger hair, a gene ontology (GO) term enrichment analysis was performed on the respective set of transcripts. This revealed that transcription-, ER-, and cell wall–related GO terms (Fig 1, S1 Data) were in accordance with the structure of the *D*. *muscipula*’s trigger hairs. Second, we classified the tissue specificity of each gene across all tissues using Shannon entropy (see Materials and methods section, S2 Data) [14]. Whereas the first analysis delivered a *p*-value for each gene in each pairwise comparison as a significance estimate, the second resulted in an entropy Q_gene|tissue_-value for each gene in each of the integrated tissues. The threshold we set to identify trigger hair–specific genes was a Q_gene|hair_-value of < 3.9 bits, comprising 1% of the total Laplace distribution area [13] (the lower the Q_gene|hair_-value, the more specific the gene is to the trigger hair tissue). Finally, these 2 methods together identified 495 genes and can thus be classified as bona fide trigger hair–specific genes (S2 Data, genes marked in red).

### Ion channels are highly specific to the trigger hair

**Fig 2:**
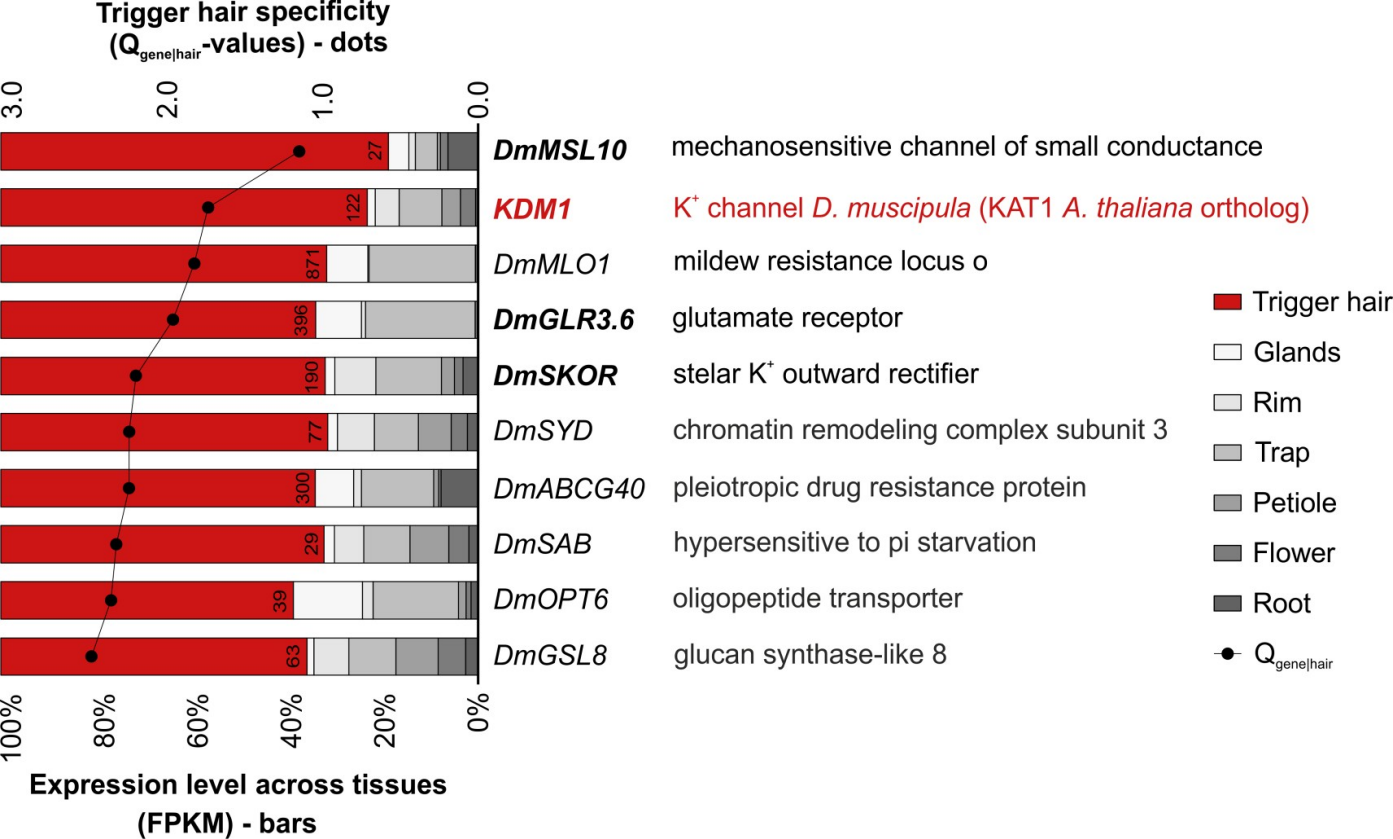
Ion channels are among the most trigger hair–specific genes. The dots represent the trigger hair Q_gene|hair_-value calculated by the Shannon entropy method for tissue specificity. A very low Q_gene|hair_-value indicates high tissue specificity. The stacked bars show the proportion to which each gene is expressed across all investigated tissues. The numbers on the red bars represent the mean FPKM expression values for the trigger hair tissue. The genes marked in bold letters represent ion channels. Shown are the top 10 highly expressed genes (trigger hair FPKM > 20) out of the bona fide trigger hair–specific genes. Genes that passed the Shannon entropy calculated Q_gene|hair_-value < 3.9 bits, comprising 1% of the total Laplace distribution area, and were at the same time trigger hair up-regulated DEGs at least 2-fold in the trigger hair compared to all the other tissues (log_2_FC > 1, BH adjusted p-value < 0.001), were considered bona fide trigger hair–specific genes. Underlying full raw dataset is provided in S2 Data. BH, Benjamini–Hochberg; DEG, differentially expressed gene; FPKM, Fragments Per Kilobase of transcript per Million mapped reads.

At the top of the above list of the most trigger hair–specific genes (S2 Data, genes marked in red), we found many electrogenic ion transporters that may be involved in the initiation of *D*. *muscipula*’s AP. Remarkably, the top 5 most trigger hair–specific genes are comprised of 4 ion channels (Fig 2, marked in bold letters) and the calcium channel–associated MLO1 [15]. As would be expected from a mechanosensitive structure, *DmMSL10*, a stretch-activated channel of the Mechanosensitive Channel of Small Conductance-Like (MSL) family, turned out to be most specific to the trigger hair (Q_gene|hair_-value = 1.13 bits). The second most specific trigger hair gene encodes a Shaker-type potassium channel (Q_gene|hair_-value = 1.70 bits). Thorough phylogenetic analysis [16] revealed that this channel groups into the K_in_-a subclade of voltage-dependent, inward-rectifying K^+^ channels and thus represents a ortholog of the *Arabidopsis thaliana* KAT1 (S2 Fig). We therefore named this *D*. *muscipula* channel KDM1 (K^+^ channel *D**ionaea* *m**uscipula* 1). Next, we identified as trigger hair specific an ortholog of the glutamate receptor *GLR3*.*6* (Q_gene|hair_-value = 1.92 bits) which, interestingly, has been shown to be involved in electrical and calcium signal propagation in *A*. *thaliana* [17], followed by *DmSKOR*, an ortholog of the *A. thaliana* stelar K^+^ outward rectifier SKOR (Q_gene|hair_-value = 2.1 bits). In order to find out in which specific region of the trigger hair these 4 channels are expressed, we analyzed the expression level of the abovementioned transporters via quantitative polymerase chain reaction (qPCR) in the 2 core parts of the hair (S3A Fig): the tip (comprising the lever which amplifies the signal) and the base (including the podium cells and the indentation zone with the sensory cells), where the bending occurs. Interestingly, this revealed that *DmMSL10* and *DmGLR3.6* are completely missing from the tip but are highly expressed in the basal part. This suggests that these channels might be involved in the trigger hair’s mechanosensation and signal transduction. Similarly, *KDM1* and the potassium outward rectifier *DmSKOR* were also more highly expressed in the base of the trigger hair than in the tip (S3B Fig).

In order to characterize the full trigger hair–specific transportome, we annotated all the transcripts via the Aramemnon plant membrane protein database [18,19]. This revealed that a number of ATP-binding cassette (ABC) transporters, P-type adenosine triphosphatases (ATPases), glutamate receptor‐like (GLR) ligand-gated cation channels, and Ca^2+^/cation antiporters (CaCA) and K^+^ uptake (KUP) transporters were also specific to the trigger hair, although these exhibited higher Q_gene|hair_-values compared to the transporters mentioned before (S4A and S4B Fig). Taken together, this led us to conclude that the highly trigger hair–specific genes *DmMSL10*, *KDM1*, *DmGLR3*.*6*, and *DmSKOR* are likely associated with the trigger hair’s sensory function and bending-induced AP generation.

### KDM1 is a trigger hair–specific voltage-dependent K^+^ channel

Next, we set out to functionally characterize the trigger hair–specific KDM1 channel (K^+^ channel *D**ionaea* *m**uscipula* 1, Dm_00004067-RA) with the help of a 3D molecular model. In line with its phylogenetic placement, the recently published *A*. *thaliana* KAT1 CryoEM structure [20] represented the best KDM1 template to generate a 3D model using either Phyre2 [21] or the SWISS-MODEL structure homology-modeling server [22]. The obtained molecular structure suggests that, like its *A*. *thaliana* ortholog, KDM1 represents a tetrameric K_V_ channel with a non-domain-swapped subunit arrangement of its voltage-sensing domains (VSDs; S1 to S4) and a central pore domain (PD; S5-P-S6), followed by a “C-linker” helix and a pseudo cyclic nucleotide-binding domain [20]. In the absence of an electrical field (0 mV), the S4 transmembrane voltage-sensing helix of these channels resides in an “up”, or depolarized, state. At the same time, a narrow constriction at the end of S6 formed by hydrophobic side chains of valine residues marks the closed inner gate of the PD. VSD activation is characterized by a hyperpolarization-driven downward movement of S4 helices. By VSD–PD coupling, the VSD downward movement leads to conformational changes in the PD. In KAT1-like channels, this process involves reorientation of the “C-linker” helices of adjacent subunits at the intracellular face of the channel, finally leading to the opening of the PD gate and ion conduction [20].

In other plant species, *KAT1* homologs are expressed in guard cells [23], where they mediate K^+^ uptake in the context of stomatal opening [24]. *KAT1* is expressed early in stomatal development [25] and is likely involved in turgor formation and guard cell expansion, processes that serve to pull the 2 embryonic guard cells apart as the stomata become functionally mature. This encouraged us to isolate stomata from *D*. *muscipula* trap and petiole tissues and analyze *KDM1* expression by qPCR. The high expression of guard cell marker genes such as *DmSLAC1* and *DmQUAC1* confirmed guard cell enrichment in this cell population [26]. *DmSKOR*, a homolog of *AtGORK*, which is also expressed in *A*. *thaliana* guard cells and known to take part in K^+^ release during stomatal closure [27,28], was found to be well expressed in *D*. *muscipula* trigger hairs, as well as being present in *D*. *muscipula* guard cells. *KDM1*, however, was not expressed in *D*. *muscipula* guard cells (S5 Fig). Thus, *D*. *muscipula* guard cells seem to operate stomatal opening independently of KDM1 and may employ other K^+^ channel subunits for this purpose (S2 Fig; [29,30]).

**Fig 3:**
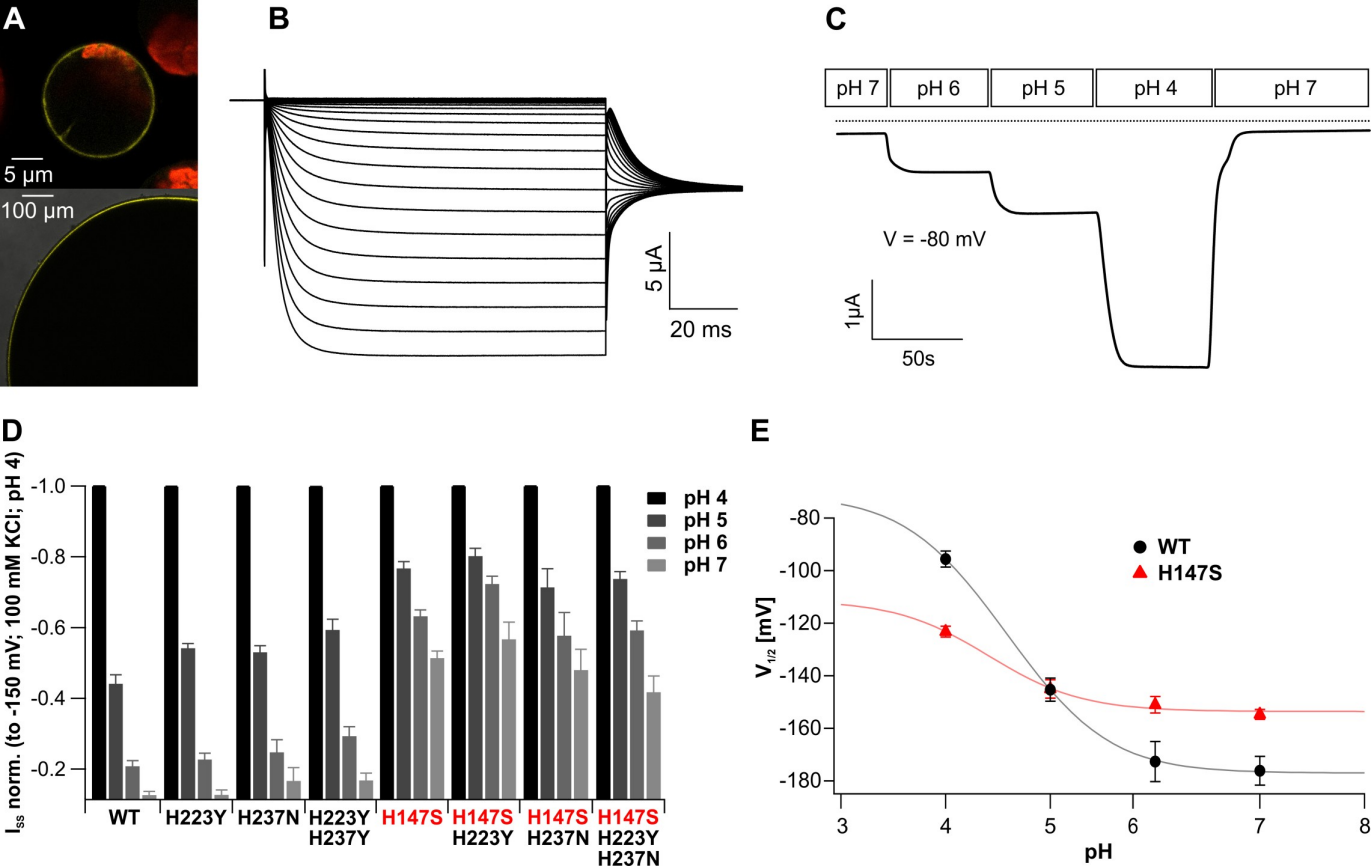
Functional properties of trigger hair–specific KDM1. (A) Localization of YFP::KDM1 fusion constructs in the PM of *A*. *thaliana* mesophyll protoplasts (top) and *X*. *laevis* oocytes (bottom). A merged picture of YFP fluorescence and the chloroplast autofluorescence is presented in the upper panel. The lower panel shows a magnified image from an enlarged section of an oocyte to demonstrate the YFP signal on the surface of the membrane. Representative images are shown. (B) KDM1 cDNA confers functional expression of hyperpolarization-activated currents in *X*. *laevis* oocytes. Currents of up to −15 μA were elicited in response to hyperpolarizing pulses from a holding potential of 0 mV in 100 mM KCl bath solution. (C) Inward K^+^ currents elicited by a KDM1 expressing *Xenopus* oocyte markedly increased upon acidification of the external solution (V_m_ = −80 mV). The dotted line represents 0 current. (D) Comparison of the I_SS_ at −150 mV of the indicated KDM1 channel mutants. I_SS_ for each mutant were normalized to −150 mV at pH 4, which displays 100% activity. Note that mutants containing H147S are less affected by an external pH change at these voltages, whereas the other histidine mutants mediate WT-like K^+^ influx (*n* ≥ 6; mean ± SE). (E) The V_1/2_ of KDM1 WT and the H147S mutant was plotted against the applied pH values and fitted as described in the Materials and methods section with the following parameters: for mutant and WT, a = 0.6 and RT/F = 25.2 mV resulted in Vs = RT/(aF) = 42mV; for WT, V_1/2_inf_ = −177.048 mV, pK_O_ = 5.1328, pK_C_ = 4.04; and for H147S, V_1/2_inf_ = −153.632, pK_O_ = 4.6285, pK_C_ = 4.1914. The V_1/2_-pH curve of the mutant is compressed compared to that of the WT pointing to fundamental alterations in the pH-sensing process. (*n* ≥ 14; mean ± SE). The full raw dataset and the statistical analysis is provided in S3 Data. PM, plasma membrane; SE, standard error; WT, wild-type; YFP, yellow fluorescent protein.

Transient expression of KDM1 N-terminally fused to yellow fluorescent protein (YFP) was assayed in isolated *A*. *thaliana* mesophyll protoplasts and confirmed that the trigger hair K^+^ channel is localized to the plasma membrane (PM) (Fig 3A, top), just like all other KAT1 channels studied before [31–33]. When YFP::KDM1 cRNA was injected into *Xenopus laevis* oocytes, fluorescence was also detected at the oocyte PM (Fig 3A, bottom), which allowed us to monitor channel activity using the Two-Electrode-Voltage-Clamp (TEVC) technique. In line with the proposed gating model, upon hyperpolarization, KDM1 mediated K^+^-dependent inward currents (Fig 3B). Increasing the external K^+^ concentration from 3 to 10, 30, and 100 mM caused increased KDM1-mediated steady state currents (I_SS_) (S6A Fig) without affecting the half-maximal activation voltage (V_1/2_; S6B Fig). Following a 10-fold change in the external K^+^ concentration, the reversal potential shifted by 54.31 ± 6.2 mV (mean ± SD; S6C Fig), which is well in line with the predicted Nernst behavior and the K^+^ selectivity of KAT1-type channels [34,35]. In contrast to *A*. *thaliana* KAT1 and *Solanum tuberosum* KST1, KDM1 activated with fast kinetics, reaching a steady state in as little as about 10 ms (t_1/2_ = 6 ms), while half-maximal amplitudes of KAT1 and KST1 were reached within t_1/2_ = 40 and t_1/2_ = 170 ms, respectively [36]. This suggests that, despite its overall sequence similarity to KAT1, KDM1 features an extraordinarily fast voltage-dependent gating (i.e., VSD movement and VSD–PD coupling).

To assess the ion selectivity of KDM1, we recorded KDM1-mediated currents in different cation solutions (K^+^, NH_4_^+^, Li^+^, Na^+^, and Rb^+^; 30 mM each at pH 4) over a voltage range of +40 mV to −190 mV. We noticed that external application of Li^+^ or Na^+^ did not lead to macroscopic currents (S6D Fig). Although reduced, inward currents could also be observed in Rb^+^ and NH_4_^+^ solutions, revealing a permeability of KDM1 for these ions as well—a finding well in line with the previous characterization of KAT1-like channels [33,37].

Besides voltage, pH plays a crucial role for the regulation of K^+^_in_-a channels, even though the molecular basis of pH sensitivity is known to differ between channels [38–41]. A stepwise increase in the extracellular proton concentration from pH 7 to 4 resulted in a rise in KDM1-mediated steady state K^+^ currents (Fig 3C and 3D, S7A Fig)—a feature shared with other members of the K^+^_in_-a subfamily [37–40]. In the process of acidification, the voltage-dependent open probability of KDM1 channels, and therefore the half-activation potential, V_1/2_, shifted from −176.1 ± 5.1 mV (mean ± SD) at pH 7 to −95.7 ± 3.1 mV (mean ± SD) at pH 4 (S7B Fig). Thus, under physiological membrane potentials, the pH sensitivity of KDM1 results in more open channels at acidic pH.

In an effort to identify pH-sensitive sites, we compared the sequence of the KDM1 with those of *A*. *thaliana* KAT1 and KAT2 as well as K^+^_in_-a channels from *S*. *tuberosum* KST1 and *Zea mays* ZmKZM1/2. This comparison identified 3 histidine residues as potential extracellular pH sensor sites specific to KDM1, namely His147, His223, and His237. According to the KDM1 model, H147 is located at the end of helix S3 (S3-helix-b), while H223 and H237 are located in the extracellular loop, linking S5 to the pore helix (S8A and S8B Fig). To test whether the acid sensitivity is associated with the histidine protonation sites present in KDM1, we generated channel mutants and analyzed them in *X*. *laevis* oocytes. Replacing this H147 with a serine, which is the corresponding amino acid in the potato KAT1-like channel KST1, led to a less pronounced pH sensitivity (Fig 3D, S8C and S8D Fig). To properly assess the effect of the H147S mutation, changes in V_1/2_ were plotted as a function of the applied pH (Fig 3E). A combined fit using Boltzmann statistics and law of mass action revealed that the overall pH sensitivity of the mutant was dampened when compared to wild-type (WT) KDM1.

The single mutations, H223Y and H237N, as well as the H223Y/H237N double mutant, showed WT-like pH sensitivity (Fig 3D). On the other hand, pH-dependent gating of any higher-order mutant comprising His147 resembled the H147S single mutant. Thus, histidine at position 147 equips the *D*. *muscipula* trigger hair–specific KDM1 channel with a pronounced pH sensitivity.

**Fig 4:**
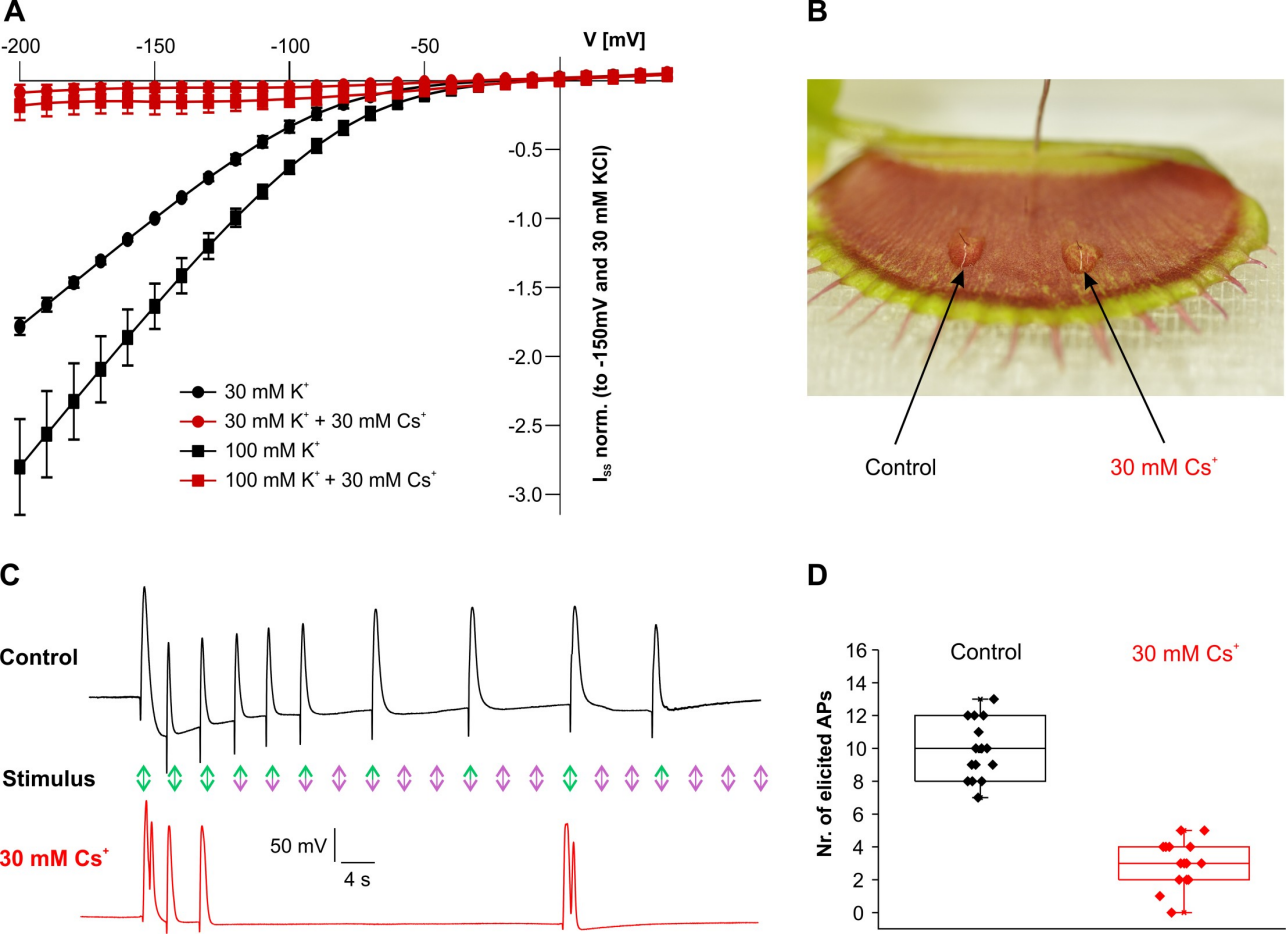
Cs^+^ blocks KDM1 and trigger hair–dependent excitability: Cs^+^ reduces the AP restoring force. (A) KDM1-mediated steady currents (normalized to −150 mV and 30 mM K^+^) at 100 mM or 30 mM external K^+^ with or without 30 mM Cs^+^. The I_SS_-V curves show inward-directed potassium currents, which ceased when Cs^+^ was applied (*n* = 17; mean ± SD). Under the same conditions, the efficiency of the cationic blocker appeared to be reduced, when Cs^+^ was replaced by TEA^+^ or Ba^2+^ (c.f. S9I Fig). (B) Trigger hairs were immersed in 1.5% low melting agarose without (left/control) and with (right) 30 mM Cs^+^ for 100 minutes. The surface potential electrode was inserted into the trap tissue for AP recordings. (C) Frequency-dependent surface potential recording of a *D*. *muscipula* trap as shown in (B). Incomplete AP block was measured after 50 minutes incubation in Cs^+^ (red). Twenty mechanical stimulations (arrows/¼ Hz) were applied to the trigger hairs. Under control conditions (black), 10 simulations were translated into APs (green arrows), while 10 (purple arrows) were not. Cs^+^ treatment reduced AP firing to just 4 (red trace). (D) Plot of the number of APs evoked in traps treated as defined in (C). Fifteen traps were each stimulated 20 times. The bottom and top of the boxes denote the first and third quartiles, respectively, the middle line is the median, and the whiskers are the most extreme values within 1.5× the interquartile distance below the first or above the third quartile. Cs^+^ treatment reduced the number of fired APs significantly (*p* = 6.0 × 10^−8^; F_1,29_ = 134.3, 1-way ANOVA, *n*  =  15). The full raw dataset and statistical analysis is provided in S3 Data. ANOVA, analysis of variance; AP, action potential; SD, standard deviation.

Another typical feature of KAT1-like K^+^_in_-a channels is their susceptibility to a block by Cs^+^ ions [42–44]. When *A*. *thaliana* guard cells are exposed to 30 mM Cs^+^, KAT1-mediated stomatal opening is impaired [44]. In line with this, when we applied 30 mM Cs^+^ in addition to 100 mM K^+^ in the external medium at pH 4, a pronounced voltage-dependent block was observed in KDM1 expressing oocytes within the physiological voltage range of −120 mV to −150 mV (Fig 4A, S9 Fig). This Cs^+^ block intensified when K^+^ and Cs^+^ were present at equimolar concentrations (Fig 4A, S9D and S9F–S9H Fig).

### Cs^+^ blocks trigger hair–dependent excitability

Knowing that KDM1 is expressed specifically in trigger hairs, we next considered whether this channel is also involved in trigger hair–dependent generation of electrical signals, making use of its Cs^+^ susceptibility. In order to test the effect of the K^+^ channel blocker on *D*. *muscipula*’s AP, the trigger hair base was covered with a film of low melting agarose containing 30 mM Cs^+^ (Fig 4B). Compared to non-treated control trigger hairs, 100 minutes after Cs^+^ application, bending of the Cs^+^-exposed trigger hair neither elicited APs nor triggered trap closure (S1 Movie). In a previous study, we showed that a trigger hair can fire about 10 APs at a stimulation frequency of 0.1 Hz before it becomes hapto-electrically silent [2]. After a refractory period, however, the same hair regains the capability to fire a new series of APs. We therefore wondered what would happen if, in the refractory period, the K^+^ gradient in the touch-sensitive cells were reestablished via KDM1-mediated K^+^ uptake? To answer this question, we tested whether Cs^+^ affects the frequency-dependent trigger hair desensitization. When stimulating control and Cs^+^-treated trigger hairs (treatment for 30 minutes) at a frequency of 0.25 Hz, 20 stimulations led to the generation of about 10 APs in the control trigger hairs, while in the presence of 30 mM Cs^+^, AP firing was found to terminate after only 4 APs (Fig 4C and 4D). Furthermore, when 5 trigger hairs within a single trap were treated with Cs^+^ agarose, they all became electrically silent, but not the untreated sixth one (S2 Movie). Covering all trigger hairs with Cs^+^ agarose completely suppressed APs resulting from trigger hair stimulation, even though the application of a more substantial force directly to the trap surface still elicited an AP (S3 Movie). This observation is well in accordance with our recent finding that exerting very strong mechanical force toward the trap blade or midrib also provoked APs [2]. Cs^+^ treatment can therefore be seen to inhibit the generation of APs from trigger hairs by blocking the activity of the K^+^ channel KDM1. On the contrary, the electrical trap network interconnecting the trigger hairs—where KDM1 is not expressed—remained fully functional (S10 Fig).

Next, we asked the question whether Cs^+^-mediated AP suppression depends on mechanical stimulation of trigger hairs. For this purpose, we impaled the mechanosensory cells in the indentation zone of the base of a single trigger hair, which served as “AP receiver.” At the same time, 2 further trigger hairs—serving as independent “AP actuators”—were treated with Cs^+^ (TH1 and TH2 in S11 Fig). Initially, mechanical stimulation of “actuator” TH1 evoked APs that could be recorded in the mechanosensory cells of the “AP receiver” trigger hair (TH3) (S11 Fig). As expected, after the Cs^+^ effect had manifested, APs triggered at the “AP actuator” trigger hair TH1 ceased. Furthermore, when now mechanically stimulating the hitherto untreated “AP actuator” trigger hair TH2, we could not elicit any further APs (S11 Fig). Trigger hair–independent heat application or trap wounding, however, still resulted in AP initiation. Taken together, these experiments indicate that Cs^+^-mediated blockage of K_in_-a channel KDM1 results in AP abrogation independent of the trigger hair’s mechanical exposure.

### How does KDM1 function affect trigger hair electrical excitability?

**Fig 5:**
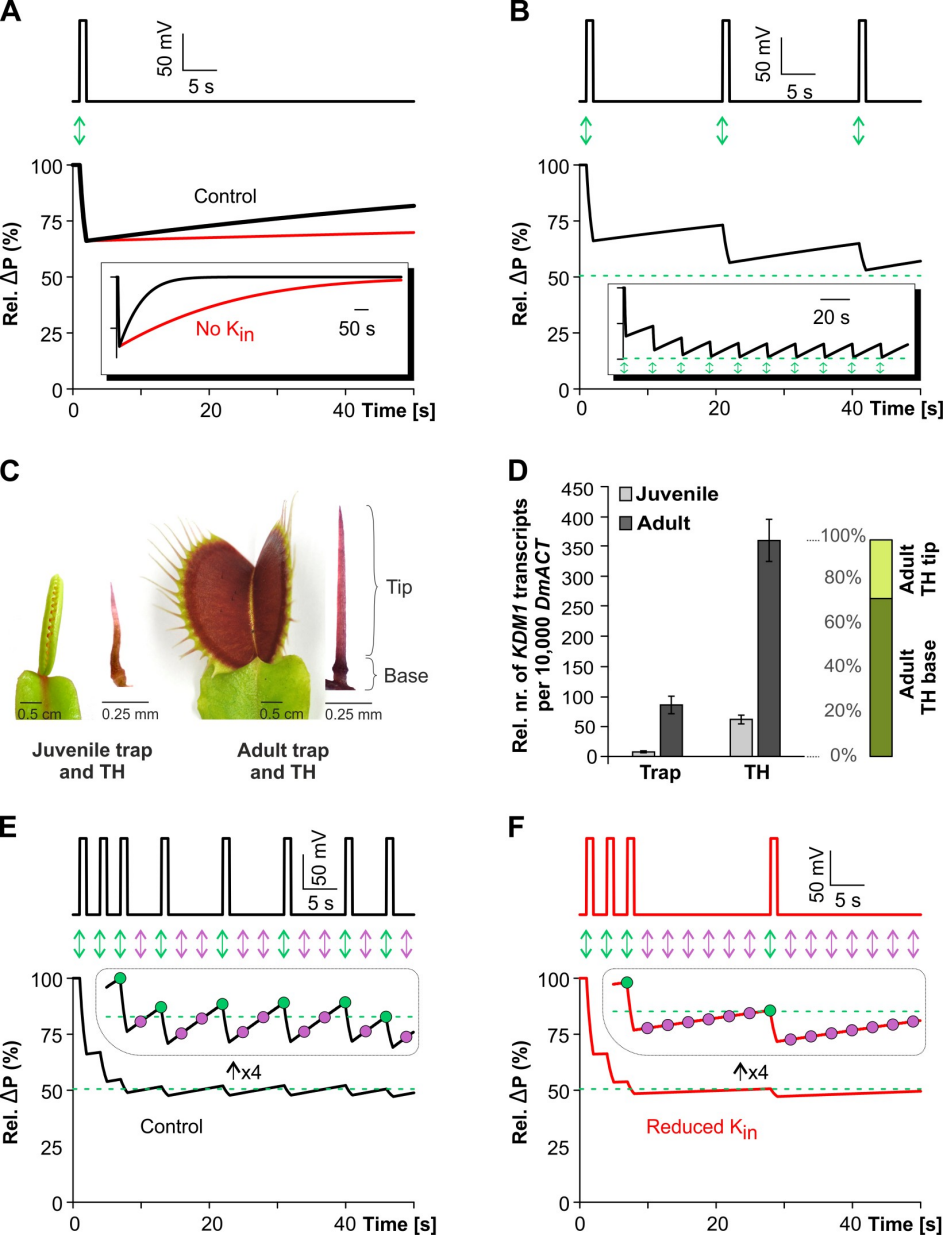
Reduced K^+^_in_ channel activity increases recovery lag time after AP firing. (A) A mechanical stimulus (green arrow) initiates an AP (upper panel) during which the accompanied ion and water fluxes reduce the cell turgor (lower panel). In the following recovery phase, the initial condition is slowly reestablished (inset shows the recovery on a larger timescale). Reduced activity of K^+^_in_ channels significantly slows down the recovery process (red curve). (B) When the THs are stimulated with low frequency (green arrows), the interval is insufficient for a full recovery of the cell. Over time, however, a new working range is established that is still above the turgor threshold (green) as seen in the inset. (C) Juvenile trap with the corresponding juvenile TH and adult trap with the corresponding adult and electrically excitable TH. (D) Normalized expression values of *KDM1* quantified by qPCR in trap and THs in juvenile and adult stages (mean normalized to 10,000 actin (± SE), *n* = 3–6; *t* test between whole adult trap and adult TH *p*-value = 0.00038). The proportion of KDM1 expression within the dissected adult TH shows a dominant (up to 70%) expression level in the base of the TH (containing the sensory cells), (*n* = 3; mean ± SE). The full raw dataset and statistical analysis is provided in S3 Data. (E) Increasing the frequency of the stimulus causes the turgor to fall below the threshold. In this condition, a mechanical stimulus may not initiate an AP and sporadic dropouts occur (purple arrows, purple dots in the enlarged view in the inset). (F) High-frequency stimulation in conditions with reduced K^+^_in_ channel activity results in larger dropout intervals. Initially, however, as long as the cell still has reserves, APs are initiated as in the control case. AP, action potential; qPCR, quantitative polymerase chain reaction; SE, standard error; TH, trigger hair.

We used a modeling approach in order to better understand the role of KDM1 for trigger hair hapto-electrics. The mechanical stimulation of trigger hairs induces a depolarization in electrically connected cells that spreads as an AP throughout the entire trap. In general, we may envision that trigger hair bending activates mechanosensing ion channels in turgescent mechanosensory cells, which then open to allow the efflux of anions and/or the influx of Ca^2+^ ions [45,46]. The resulting membrane potential depolarization, in turn, activates voltage-activated K^+^_out_ channels, like DmSKOR [47,48]. It has been observed that this depolarized state lasts about 1 second and is achieved when the anion (A^−^) efflux, possibly via DmMSL10 or Ca^2+^-activated anion channels [46], is electrically compensated by an accompanying K^+^ efflux. During the hyperpolarized recovery phase of the AP, the PM H^+^-ATPase energizes the resumption of K^+^ and A^−^ until the initial equilibrium is restored [49]. According to the model (see Materials and methods section), K^+^/A^−^ fluxes during the AP not only change the concentration of both ions within the cell ([*K*^*+*^]_*cell*_) and in the apoplast ([*K*^*+*^]_*apo*_) but also at the same time affect the osmotic pressure difference (ΔP) across the membrane, which at equilibrium in the resting state is about 5 bar. In a simulation (parameters given in the Materials and methods section), the combined efflux of K^+^ and A^−^ during the depolarization phase of a single AP was sufficient to reduce [*K*^*+*^]_*cell*_ by approximately 30%, to increase [*K*^*+*^]_*apo*_ by approximately 300%, and to reduce the osmotic pressure (ΔP) by approximately 33% in the time span of 1 second (Fig 5A, control, black). Taking into account slightly slower uptake rates for K^+^/A^−^ influx into the cell during repolarization, the system recovered more slowly, eventually reattaining the original starting conditions via an increase of [*K*^*+*^]_*cell*_, a decrease of [*K*^*+*^]_*apo*_, and an increase of Δ*P* (Fig 5A, inset rel.ΔP). When a trigger hair was stimulated repetitively with a low frequency (0.05 Hz), an AP was initiated on each occasion (Fig 5B). As seen in Fig 5B inset, the intervals between the stimuli were not long enough for a full recovery of the cells, with them instead adjusting to a new operating range of 51% to 61% of their resting level. In these conditions, the turgor was always above a critical pressure threshold, which was set to approximately 50% in the simulation (green dashed lines in Fig 5B). Below this turgor threshold, movement of the trigger hair did not result in the activation of the mechanosensitive channels. Indeed, when monitoring trap development, fully erect trigger hairs were only found in the final maturation stage (Fig 5C). *KDM1* expression was weak in immature trigger hairs (Fig 5D), and touching the flaccid trigger hairs of juvenile traps did not electrically excite the immature capture organ. A subcritical turgor state was achieved by reducing the recovery time, i.e., increasing the stimulus frequency (Fig 5E). Initially, the fully charged cell possessed sufficient capacity to initiate an AP upon a given mechanical stimulus. After a few stimuli, however, the turgor dropped below the critical threshold, and ongoing stimulation was insufficient to activate the mechanosensitive cation channels (Fig 5E, purple arrows). The dropout-based additional lag time, however, allowed the cell to recover turgor above the threshold and to fire an AP upon the next trigger hair stimulation (Fig 5E, green arrows). This prediction is in line with previously observed dropouts in AP patterns upon high-frequency stimulation [2] and shows that the applied model is well suited to explain experimentally observed AP phenomena. The model further suggests that the observed dropouts during continuous high-frequency mechanical stimulation can be explained by a turgor-dependent impairment of mechanoelectrical signal transmission.

Since potassium homeostasis at the PM is established by the dynamic interplay of H^+^-ATPases, K^+^ channels, H^+^/K^+^ symporters, and H^+^/K^+^ antiporters [49–51], we next asked the model what would happen if KDM1 activity was blocked (e.g., by Cs^+^ application). Here, we found that if the activity of K^+^ uptake channels was reduced in this transporter network, the energy costs for K^+^ uptake increased. This is because uptake of a K^+^ ion via a K^+^ channel needs the ATP-driven extrusion of a proton (hydrolysis of 1 ATP molecule), while uptake via a H^+^/K^+^ antiporters would double the energy costs [50,52]. Consequently, K^+^ uptake, and thus turgor formation, slows down when K^+^ uptake channels are inhibited [30,53]. Assuming a 5-fold reduction of K^+^ uptake channel activity under Cs^+^ blocking conditions in the simulation, an AP evoked by a single trigger hair stimulation induced the same turgor changes as under control conditions. The time span for full recovery after the AP, however, was 5 times longer under conditions of reduced K^+^_in_ activity (Fig 5A, red). High-frequency, repetitive stimulations with blocked K^+^_in_ channel activity initially elicited control-like AP series. At some point, however, when the osmotic pressure difference fell below the critical threshold, the recovery time was significantly extended. This resulted in longer dropouts in the AP pattern (Fig 5F). Exactly the same phenomenon was observed experimentally when exposing trigger hair cells to extracellular Cs^+^ (Fig 4).

## Discussion

Previous studies on the biomechanics of the *D. muscipula* trigger hair showed that this specialized structure is designed to efficiently translate minimal mechanical stimuli into APs that trigger rapid trap closure [2]. To gain molecular insights into the unique functional inventory of this hapto-electric organ, we analyzed the trigger hair transcriptome by comparing it with the transcriptomes of other *D*. *muscipula* tissues and organs. We thereby identified 4 AP-related electrogenic ion channels that were highly specific to the trigger hair: *DmMSL10*, *KDM1*, *DmGLR3.6*, and *DmSKOR*.

Touch activation of the trigger hair that results in depolarization of cells in the indentation zone and AP initiation could be brought about by the highly trigger hair–specific DmMSL10 [46]. MSLs have previously been characterized as mechanosensitive anion channels in noncarnivorous *A*. *thaliana* plants [46,54]. The molecular nature of the ion channels involved in the depolarizing phase of *D*. *muscipula*’s trigger hair-derived AP is not known, but experimental data from *Chara braunii* suggest that Ca^2+^ influx and Ca^2+^-activated chloride channels are involved [55–58]. The repolarizing phase, however, similar to animal APs, is very likely driven by K^+^ efflux via the depolarization-activated potassium channel DmSKOR, which also appears to be specific for the trigger hair transportome [48]. In the final phase of the AP, AHA-type H^+^ pumps transiently hyperpolarize the PM and acidify the cell wall [49,59].

The extracellular space of the trigger hair’s sensory cells located in the indentation zone is confined. From this compartment, the loss of potassium ions during DmSKOR-mediated repolarization of the membrane potential must be compensated during the refractory period, which could be accomplished by KDM1 activity. Specifically, this trigger hair K^+^ channel increases its open probability with extracellular acidification. Thus, the driving force for K^+^ influx and the probability of the KDM1 channel opening is highest during the hyperpolarization and acidification phase of the AP. Under such conditions, KDM1 can shuttle K^+^ back into the sensory cells in order to reestablish the trans-plasma membrane K^+^ gradient and cell turgescence. Cs^+^, meanwhile, blocks KDM1, which prevents AP generation when trigger hairs are stimulated, in our view because the K^+^ gradient in the indentation zone cells, and consequently turgor pressure, has not yet been rebuilt. It is important to note, however, that Cs^+^ inhibits the AP generation only in the treated trigger hair, while the electrical network of the entire trap remains fully functional.

Both sensory cells in the indentation zone, and the terminal cells within the trigger hair lever, are characterized by an elevated potassium content, which is at least 3 times higher than in the surrounding epidermal cells [60], indicating the importance of an osmotic component in the system. Given that cell elongation in plants is associated with cell wall acidification, K^+^ uptake and turgor formation (e.g., in the *Z*. *mays* coleoptile [61,62]), KDM1 activation at low external pH might therefore be crucial for K^+^ uptake and turgor formation in the mechanosensory cells of the trigger hair. Since the AP originates among the cells of the peripheral ring in the indentation zone of the trigger hair base [5], it can be assumed that the trigger hair, and especially the mechanosensory cells in the trigger hair base, have to reach a fully turgescent stage to be able to translate touch into *D*. *muscipula*’s characteristic all-or-nothing AP. In line with this hypothesis, when we separated the trigger hair tip from its base, KDM1 was indeed found to be more highly expressed in the podium fraction (Fig 5D, S3 Fig). This notion is supported by simulation experiments, underlining the contribution of KDM1 activity to turgor formation and electrical signaling in *D*. *muscipula*’s trigger hairs.

## Materials and methods

### Plant material and tissue sampling

*D*. *muscipula* plants were purchased from CRESCO Carnivora and grown in plastic pots at 22°C with a 16:8-hour light:dark photoperiod. For the RNA sequencing (RNA-seq) experiments, 3 biological replicates per tissue were prepared. The glands were isolated by gently abrading the gland complexes from the trap surface, in which case 8 traps were needed per replicate. The trigger hairs were gently removed using tweezers under a binocular. A total of 300 trigger hairs from about 50 traps were collected for 1 replicate. Harvested tissues were immediately frozen in liquid nitrogen, while for the trigger hairs and glands, RNAlater (Sigma-Aldrich, Taufkirchen, Germany, R0901-100ML) was used to maintain RNA integrity at 4°C until isolation.

For the qPCR expression measurements of genes of interest, the trigger hair was dissected into 2 parts: The basal part and the tip were separated using a scalpel and a binocular and immediately stored in RNAlater (Sigma-Aldrich, R0901-100ML) at 4°C until processed. Since this was a different experiment to the one processed for RNA-seq analysis, entire/undissected trigger hairs were also collected as a control. The qPCR expression analysis for the whole trigger hairs needed between 300 and 600 trigger hairs for 1 replicate, while for the separate tip and the base parts, between 880 and 940 trigger hairs were needed to extract enough RNA for 1 replicate. Three replicates were used in total.

For preparation of guard cell–enriched petiole and trap samples, midribs were removed, and guard cells were isolated by blender cycles (90 seconds each) in ice-cold deionized water with additional crushed ice and subsequently filtered through a 210-μm nylon mesh. For a detailed description, see [63]. The remaining guard cell–enriched fraction of traps and petioles was immediately frozen in liquid nitrogen. Neutral red staining indicated that the viable cells in the preparations were guard cells.

### RNA isolation

For the RNA-seq experiments, the trigger hair tissue was stored in RNAlater (Sigma-Aldrich,R0901-100ML) at 4°C until further processing. The samples were then centrifuged, and the RNAlater solution was discarded. RNA-extraction buffer (2% CTAB, 2% polyvinylpyrrolidone K 25 (PVP), 100 mM TRIS/HCl at pH 8.0, 25 mM Na-EDTA at pH 8.0, 2 M NaCl, with 2.5% [v/v] 2-mercaptoethanol added immediately before use) was added to the plant tissue, and the RNA isolation protocol was followed as described before for other tissues (petiole, flower, root, rim, trap, and glands) [8]. RNA quantity and quality were checked by capillary electrophoresis (Experion automated electrophoresis system and Experion RNA high sense analysis kit, Bio-Rad Laboratories, Feldkirchen, Germany). All replicates had a good quality (RNA quality indicator (RQI) of trigger hair tissue replicates: 9.7, 7.0, and 8.5) and were further processed by a commercial provider (LGC Genomics, Berlin, Germany).

For qPCR analyses, RNA was isolated from each sample using the NucleoSpin Plant RNA extraction kit (Macherey-Nagel, Düren, Germany) according to the manufacturer’s instructions and in combination with Fruit-mate for RNA Purification solution (Takara Bio Europe SAS, Saint-Germain-en-Laye, France). Briefly, 100 mg of powdered plant material was thoroughly mixed with 350 μl of Fruit-mate solution for 1 minute. Following 10 minutes of centrifugation (20,000 rcf) at 4°C, the supernatant was mixed with the lysis buffer provided by the kit (RAP), together with 3.5 μl TCEP (Tris(2-carboxyethyl)phosphine hydrochloride, 0.5M, pH 7, Sigma-Aldrich). Apart from this, the kit manufacturer’s instructions were followed except for the DNA digestion, which was performed in a separate step after the RNA isolation. For 1 μg of RNA, 3 μl DNase Buffer (10x Reaction Buffer with MgCl_2_ for DNase, Thermo Fisher Scientific, Darmstadt, Germany), 0.5 μl DNase inhibitor (RiboLock RNase Inhibitor 40 U/μl, Thermo Fisher Scientific), and 1 μl DNase I (DNase I, RNase free 1000 units (1U/μl), Thermo Fisher Scientific) were mixed in a final volume of 30 μl and incubated at 37°C for 30 minutes. Next, the DNA-free RNA was precipitated in isopropanol overnight at −20°C, together with 1% glycogen (RNA Grade (20 mg/ml), Thermo Fisher Scientific), 10% NH_4_-Ac (5 mM in EDTA), 60% Isopropanol (2-Propanol AppliChem, Darmstadt, Germany), and water up to a final volume of 100 μl. The samples were washed using 70% Et-OH, centrifuged at 4°C for 20 minutes, and the resulting pellet was dried at 37°C and resuspended in water (DEPC, AppliChem).

The RNA was transcribed into cDNA using the M-MLV Reverse Transcriptase (RNase H- Point Mutant, Promega, Walldorf, Germany). qPCR was performed using a Realplex Mastercycler system (Eppendorf, Hamburg, Germany) and ABsolute QPCR SYBR green capillary mix (Thermo Fisher Scientific). Quantification of the actin transcript *DmACT1* (GenBank: KC285589, Dm_00017292-RA) and transcripts for *D*. *muscipula* genes of interest was performed by real-time PCR as described in [8]. *D*. *muscipula* transcripts were normalized to 10,000 molecules of *DmACT1*. S1 Table lists the qPCR conditions and primers used.

For the guard cell samples, the standard procedure described above was followed, except that 750 μl of Fruit-mate solution were used and, after the centrifugation step, 500 μl of the supernatant was mixed with 500 μl of lysis buffer. For the dissected trigger hair samples that were stored in RNAlater, the Fruit-mate solution step was omitted, and the lysis buffer was added to the plant material after centrifugation and removal of the RNAlater solution.

### RNA sequencing data processing

All samples were sequenced on an Illumina HighSeq2000 platform. The RNA-seq data of the petiole, flower, root, rim, trap, and gland tissues were used from already published data (see [8]) with an average of 200 million total paired reads per tissue. Trigger hair RNA-seq data were generated later on a separate flow cell with a total of 196 million high-quality paired reads (S2 Table). Being fully aware of the need to account for different sequencing runs, all data were reanalyzed together and normalized within the differential expression analysis as described below.

All data have been deposited in GenBank’s Sequence Read Archive (SRA); sequencing reads can be downloaded under the Project ID PRJEB38423.

Prior to transcriptome gene expression quantification, the RNA-seq paired-end reads (more than 50 million read pairs per replicate; 3 replicates per tissue sample) were quality checked using FastQC (High Throughput Sequence Quality Check Report version 0.11.5). Since all the replicates showed high-quality reads (S2 Table), the reads were further mapped to the indexed *D*. *muscipula* assembled genome [13] using STAR Aligner (version 2.5.0a) with the default options [64]. The quantification of the mapped reads was performed using HTseq (version 0.11.0 [65]) with the following options: -f bam -r pos -s no -i transcript_id. For each gene, the number of raw counts for each tissue was obtained. For differential expression analysis, the R (version 3.4.4) DEseq2 package was used with the DEseqDataSetFromHTSeqCount function [66], obtaining a BH adjusted *p*-value for each of the pairwise comparisons (the trigger hair was compared to each of the other tissues; raw DEseq2 output can be found in S1 Data). A gene was considered to be a DEG when it exhibited a BH adjusted *p*-value < 0.001 within the pairwise comparison. The log_2_FC (log_2_FoldChange) values of each DEG passing the threshold BH adjusted *p*-value < 0.001 are shown in S2 Data.

The PCA plot was generated with DEseq2 R (version 3.4.4) package from rlog (regularized logarithm) values of each tissue replicate (data can be found in S4 Data). The pairwise correlation plot based on normalized Fragments Per Kilobase of transcript per Million mapped reads (FPKM) expression values was done using corrplot R (version 3.4.4) package [67], showing Pearson correlation coefficient value for each pairwise comparison (data can be found in S4 Data).

The intersection plot of DEGs in the trigger hair compared to the other tissues (trap, rim, glands, petiole, flower, and root) was generated using UpSetR R (version 3.4.4) package [68].

### Gene ontology terms enrichment analysis

The GO terms were assigned with Interproscan (version 5.25–64.0) using the--goterms parameter [13]. Further GO terms enrichment studies were done using Ontologizer (version 2.1) applying the Parent–Child–Union calculation method and the BH method for *p*-value correction [69]. Only GO terms with a BH corrected *p*-value < 0.05 were considered to be significantly enriched (S1 Data).

### Tissue specificity analysis

In order to identify trigger hair–specific genes, we applied 2 complementary methods. We combined the Shannon entropy method for tissue specificity [13,14], with differential expression analysis. The Shannon entropy method calculates an overall tissue specificity value for each gene, called H_gene_-value, across all analyzed tissues. A small H_gene_-value means that the gene is expressed in very few tissues. For each tissue, a Q_gene|tissue_-value is calculated, indicating in which of the analyzed tissues the expression is the highest. This method allows us to easily classify transcripts from highly specific (very low Q_gene|tissue_-values) to ubiquitous (very high Q_gene|tissue_-values) within a specific tissue of interest, which in our case was the trigger hair tissue. For a stricter variation between the replicates, intersection analysis of DEGs was used. Genes that passed the Shannon entropy calculated Q_gene|hair_-value < 3.9 bits, comprising 1% of the total Laplace distribution area and were at the same time up-regulated at least 2-fold in the trigger hair compared to all the other tissues (log_2_FC > 1, BH adjusted *p*-value < 0.001), i.e., genes passing the thresholds of both methods were considered bona fide trigger hair–specific genes (S2 Data, genes marked in red).

### Gene annotation

The predicted FASTA sequences of the *D*. *muscipula* transcriptome [13] were functionally annotated with the default BLAST cutoff using Mercator v3.6, which is specialized for functional annotation of plant “omics” data [70].

To further characterize the *D*. *muscipula* transportome, all transcripts were blasted against the Aramemnon plant membrane protein database [18,19].

### Phylogenetic tree construction

The phylogenetic tree was generated by the pipeline described in Dreyer and colleagues [16] using the following species with publicly available transcriptome or genome data: *Aldrovanda vesiculosa*, *Amborella trichopoda*, *Aquilegia coerulea*, *A*. *thaliana*, *Beta vulgaris*, *Cephalotus follicularis*, *D*. *muscipula*, *Drosera adelae*, *Drosera spatulate*, *Genlisea aurea*, *Mimulus guttatus*, *Nelumbo nucifera*, *Nepenthes alata*, *Nymphaea colorata*, *Oryza sativa*, *Populus trichocarpa*, *Rhododendron delavayi*, *Sarracenia purpurea*, *Solanum lycopersicum*, *Utricularia gibba*, and *Vitis vinifera*.

### *Xenopus* oocyte preparation

The investigations into the KDM1 transport function were performed in oocytes of the African claw frog, *X*. *laevis*. Mature female *X*. *laevis* frogs were kept at 20°C with a 12/12-hour day/night cycle in dark gray 96 l tanks (5 frogs/tank). The frogs were fed twice a week with floating trout food (Fisch-Fit Mast 45/7 2mm, Interquell GmbH, Wehringen, Germany). Tanks were equipped with 30-cm long polyvinyl chloride (PVC) pipes with a diameter of around 10 cm as hiding places for the frogs. The water was continuously circulated and filtered using a small aquarium pump. For oocyte isolation, mature female *X*. *laevis* frogs were anesthetized by immersion in water containing 0.1% 3-aminobenzoic acid ethyl ester. Following partial ovariectomy, stage V or VI oocytes were treated for 1.5 hour with 0.14 mg/ml collagenase I in Ca^2+^-free ND96 buffer (10 mM HEPES pH 7.4, 96 mM NaCl, 2 mM KCl, 1 mM MgCl_2_,). Subsequently, the oocytes were washed with Ca^2+^-free ND96 buffer and kept at 16°C in ND96 solution (10 mM HEPES pH 7.4, 96 mM NaCl, 2 mM KCl, 1 mM MgCl2, 1mM CaCl_2_) containing 50 mg/l gentamycin.

### Ethics statement

For experiments with *X*. *laevis* frogs, we follow the 3R-Principle (Replacement, Reduction, Refinement). Concerning the animal welfare and housing, we strictly follow the guidelines of the Federation of European Laboratory Animal Science Associations (FELASA) and the “Tierschutz-Versuchstierverordnung” (TierSchVersV), which involves regular inspections of our animal facility by the animal welfare officer every 3 months. Permission for keeping and surgery of *X*. *laevis* frogs exists at the Julius-von-Sachs Institute of the University of Würzburg and is registered at the government of Lower Franconia (reference number 55.2-2532-2-1035). For partial ovariectomy, mature female *X*. *laevis* frogs are anesthetized. Following the surgery, the health of *X*. *laevis* frogs are regularly monitored, and the physical/health condition is registered in score sheets.

### Cloning of KDM1

Searching the *D*. *muscipula* genome revealed a coding sequence related to KAT1-like potassium channels. PolyA-mRNA was isolated from 10 μg of total RNA using Dynabeads (Invitrogen by Thermo Fisher Scientific, Darmstadt, Germany) according to the manufacturer’s instructions. cDNA was generated from *D*. *muscipula* trigger hair mRNA. The cDNA of KDM1 was amplified using gene-specific oligonucleotide primers (forward: 5′-ATG TCA TTT TCT CGT ATG AAA CAG-3′; reverse: 5′-CTA GAG AAA GAA CAG ATG ATC GCC-3′) and the Advantage cDNA polymerase mix (Clontech by Takara Bio Europe SAS, Saint-Germain-en-Laye, France). The full-length clone was inserted into pGEM-T Easy using the pGEM-T Easy Vector System I (Promega). For heterologous expression in *X*. *laevis* oocytes, the cDNA generated from KDM1 was cloned into oocyte expression vectors (based on pGEM vectors) using the advanced uracil excision-based cloning technique as described [71]. For functional analysis, cRNA was prepared using the mMessage mMachine T7 Transcription Kit (Invitrogen by Thermo Fisher Scientific, Darmstadt, Germany). For oocyte electrophysiological experiments, 25 ng of KDM1 cRNA was injected and expressed for between 1 and 2 days at 16°C in ND96 solution containing gentamycin.

### Generation of KDM mutants

KDM1 mutants were generated by PCR and a USER fusion method as described above [71]. The following primers were used: KDM H147S fw: 5′-AGT CTC TCG GUC TCC TGG TTT CAG-3′, KDM H147S rev: 5′-ACC GAG AGA CUG CAG TGG CAC TGT TGA AC-3′, KDM H223Y fw: 5′-ACG TAC CCU GAT CCA AAG AAA ACT TG-3′, KDM H223Y rev: 5′-AGG GTA CGU ATC TGC TAA CAG ATA ATT AAT GC-3′, KDM H237N fw: 5′-ATC CAA ATU TTA AAG AAG AAG GTC TGT GG-3′, and KDM H237N rev: 5′-AAT TTG GAU TTA CTG CAC CAA TCC-3′.

### Oocyte recordings

Basic solutions for recording the electrophysiological properties of KDM1 expressing oocytes contained 100 mM KCl, 1 mM CaCl_2_, 1 mM MgCl_2_, 1 mM LaCl_3_ and 10 mM citrate/Tris (pH 4 and pH 5) or 10 mM MES/Tris (pH 6 and pH 7). When the potassium concentration deviated from 100 mM, the ionic strength was adjusted with LiCl. For the selectivity measurements, potassium salt was replaced by the indicated monovalent cation chloride salts. The blocking substances were also used as chloride salts and added in concentrations as indicated in the figures. The voltage-clamp recordings on *X*. *laevis* oocytes were performed as described [72,73]. In order to analyze the steady state currents (I_SS_), test pulses (50-ms duration) were applied in −10 mV decrements from +40 to −200 mV based on a holding potential of 0 mV. The I_SS_ were measured at the end of the test pulses. Tail currents (I_tail_) were measured at the beginning of the pulses after the capacitive currents. The determination of the relative open probability was performed as described [73] with a constant voltage pulse of −200 mV subsequent to different test pulses. Due to a very high variance in the current amplitude of individual oocyte cells, the currents were normalized to conditions as described in the figure legend. Thereby, the normalization was performed for each cell and thus, each measuring point is in relation to a value normalized to 1. After this normalization of each single cell, the resulting mean values were plotted against the applied voltages.

In order to analyze the pH dependence of KDM1 and the H147S mutant, we used a model solidly based on the Boltzmann statistics and the law of mass action that has been presented by [74]. In this model, the open probability of a channel can be described by a simple Boltzmann function (po = 1/(1+exp(a*F/RT*(V-V1/2))). The parameter V_1/2_ (voltage, at which 50% of the channels are open) depends on the external pH according to the equation V_1/2_ = V_1/2_inf_−RT/(a*F) * ln[(10^(pH) + 10^(pK_C_))/(10^(pH) + 10^(pK_O_))].

Data were analyzed using Igor Pro and Origin Pro 9.0G.

### Localization studies

*A*. *thaliana* mesophyll protoplasts were isolated and transiently transformed as described in [72,75]. For transient protoplast expression of KDM1 and the localization in *X*. *laevis* oocytes, the cDNA was cloned into the plant expression vector pSAT (driven by the UBQ10 promoter) and oocyte expression vectors (based on pGEM vectors), respectively, via the advanced uracil excision-based cloning technique [71]. Thereby, KDM1 was fused N-terminally to the vector-based YFP for localization studies. The measurements were performed on a Leica SP5 confocal laser scanning microscope (Leica Microsystems CMS GmbH, Mannheim, Germany) using the water immersion objective lens Leica HCX IRAPO L25×/0.95W for confocal imaging. YFP fusion proteins were excited with a 514-nm diode laser, and YFP fluorescence emission was detected between 530 and 560 nm. In *A*. *thaliana* protoplasts, the chlorophyll autofluorescence was detected at 630 to 750 nm.

### Structural alignment

For sequence alignment, the following KAT1-orthologs were used: AtKAT1 (PDB structure ID 6V1Y, accession number At5g46240), AtKAT2 (At4g18290), StKST1 (Q41461_SOLTU), ZmKZM1 (GRMZM2G081666), and ZmKZM2 (GRMZM2G093313). The structural alignment of KDM1 was performed with the recently published cryogenic electron microscopy (cryo-EM) structure of its *A*. *thaliana* ortholog KAT1 [20]. Protein alignment was performed using MAFFT [76], and the KDM1 model was obtained using SWISS-Model [22] and analyzed using PYMOL [77].

### Membrane potential measurements

For membrane potential recordings, the lobe of a cut trap was glued to the base of a chamber and left to recover (30 minutes) in a plant standard solution containing 1 mM KCl, 1 mM CaCl_2_, and 10 mM MES, adjusted with Bis-Tris-Propane buffer to pH 6. Trigger hairs were stimulated by a micromanipulator bending the trigger hair at given time points.

To quantify the Cs^+^-block in *D*. *muscipula*, we used surface electrodes measuring the extracellular potential of the trap tissue. One silver electrode was impaled into the trap surface, while the reference electrode was put into the wet soil or the petiole. Electrical signals were amplified 100× and recorded with PatchMaster software (HEKA Elektronik GmbH, Lambrecht, Germany). Trigger hair stimulation or wounding of the trap tissue (i.e., strong pressure on the trap tissue) was applied at the given time points.

For impalements, microelectrodes from borosilicate glass capillaries with filament (Hilgenberg, Malsfeld, Germany) were pulled on a horizontal laser puller (P2000, Sutter Instruments by Science Products GmbH, Hofheim, Germany) and filled with 300 mM KCl and connected via an Ag/AgCl half-cell to a headstage (1 GΩ, HS-2A, Axon Instruments). Tip resistance was about 30 MΩ, while the input resistance of the headstage was 1,013 Ω. The reference electrode was also filled with 300 mM KCl. An IPA-2 amplifier (Applicable Electronics, Sandwich, USA) was used, and the cells were impaled by an electronic micromanipulator (NC-30, Kleindiek Nanotechnik, Reutlingen, Germany). An intact “receiver” trigger hair was impaled into the podium cells, and the “actuator” trigger hairs were bent to evoke APs at given time points. To ensure that the “actuator” trigger hairs were toxified by Cs^+^, the trap tissue was cut alongside the trigger hairs. CsCl was applied in the concentrations indicated in the figures.

### Mechanistic model for the initiation of action potentials at the base of trigger hairs

The mechanical stimulation of trigger hairs induces a depolarization in connected cells that spreads as an AP throughout the entire trap. Non-turgescent trigger hair cells, e.g., during development, are incapable of initiating an AP. Therefore, it is reasonable to assume that trigger hair bending activates mechanosensing cation channels in turgescent cells, which open and allow the influx of Ca^2+^ ions. This depolarizes the membrane. The depolarization during an AP takes about 1 second. During this time, K^+^ flows out of the cell via voltage-activated K_out_ channels. The K^+^ flux must be electrically compensated by an accompanying anion efflux, otherwise the membrane potential would not remain depolarized for a longer period, but would immediately repolarize. In the mathematical description of our mechanistic model, we described the K^+^ flux rate via the K_out_ channels as

$$j_{K,efflux}=a_{K}∙\left(V_{excited}-\frac{RT}{F}∙\ln\left(\frac{{\left[K^{+}\right]}_{apo}}{{\left[K^{+}\right]}_{cell}}\right)\right)∙trigger$$ (1)

Here, *V*_*excited*_ is the membrane voltage in the excited state, *R*, *T*, and *F* have their usual meaning (*RT*/*F* ≈ 25 mV), and [*K*^*+*^]_*apo*_ and [*K*^*+*^]_*cell*_ denote the potassium concentrations in the apoplast and the cell, respectively. The parameter *a*_*K*_ has the unit mol/(mV·s). The binary parameter *trigger* can take on the value 1 or 0. It is 1 when the cell is excited/depolarized and 0 when it recovers. For simplicity, we assume a counterion to be a monovalent anion, which therefore flows with the same flux rate:

$$j_{A,efflux}=j_{K,efflux}$$ (2)

During the hyperpolarized recovery phase, the H^+^-ATPase energizes the resumption of K^+^ and A^−^ until the initial equilibrium is achieved again. A simple way to describe this K^+^ uptake without introducing unnecessary free parameters is

$$j_{K,influx}=a_{K}∙\beta ∙\left(V_{rest}-\frac{RT}{F}∙\ln\left(\frac{{\left[K^{+}\right]}_{apo}}{{\left[K^{+}\right]}_{cell}}\right)+V_{0}\right)∙(1-trigger)$$ (3)

*V*_*rest*_ is the membrane voltage in the recovery state, and *V*_*0*_ is a parameter that determines the resting condition. The influx stops when the concentration gradients are established again, and *E*_*K0*_ = *V* + *V*_*0*_ is reached. The dimensionless parameter β is in the range 0 < β < 1 because the uptake process consumes energy from ATP hydrolysis and is slower than the downhill K^+^/A^−^ release during the efflux phase. The uptake process should reset the cell to its original status. It is therefore straightforward to assume that the flux rate of the counter anion here is approximately identical to the K^+^ flux rate:

$$j_{A,influx}=j_{K,influx}$$ (4)

Other assumptions would only increase the mathematical complexity without changing the results qualitatively. The K^+^/A^−^ fluxes change the concentration of both ions within the cell and in the apoplast. In the small time interval *dt*, these changes are determined by

$$d{\left[K^{+}\right]}_{cell}=-\frac{\left(j_{K,efflux}+j_{K,influx}\right)}{{Vol}_{cell}}∙dt$$ (5)

$$d{\left[K^{+}\right]}_{apo}=+\frac{\left(j_{K,efflux}+j_{K,influx}\right)}{{Vol}_{apo}}∙dt$$ (6)

$$d{\left[A^{-}\right]}_{cell}=-\frac{\left(j_{K,efflux}+j_{K,influx}\right)}{{Vol}_{cell}}∙dt$$ (7)

$$d{\left[A^{-}\right]}_{apo}=+\frac{\left(j_{K,efflux}+j_{K,influx}\right)}{{Vol}_{apo}}∙dt$$ (8)

with the volumes *Vol*_*cell*_ and *Vol*_*apo*_ (= α·*Vol*_*Cell*_) of the cell and the surrounding apoplast, respectively. α describes by how much the extracellular space is larger than the cell. The concentration changes affect the osmotic pressure difference across the membrane, Δπ. Assuming a relatively rapid equilibration of the connected changes in the gradient of the water potential, the concentration changes thus result in alterations of the hydrostatic pressure difference across the membrane, Δ*P*:

$$d\Delta P=d\Delta \pi =RT∙(d{\left[K^{+}\right]}_{cell}+d{\left[A^{-}\right]}_{cell}-d{\left[K^{+}\right]}_{apo}-d{\left[A^{-}\right]}_{apo})$$ (9)

$$=-\frac{2RT}{{Vol}_{cell}}∙\frac{\alpha +1}{\alpha }∙(j_{K,efflux}+j_{K,influx})∙dt$$

$$=-\frac{2RT∙a_{K}}{{Vol}_{cell}}∙\frac{\alpha +1}{\alpha }∙$$

$$\left((V_{excited}-\frac{RT}{F}∙\ln\left(\frac{{\left[K^{+}\right]}_{apo}}{{\left[K^{+}\right]}_{cell}}\right))∙trigger+\beta ∙(V_{rest}-\frac{RT}{F}∙\ln\left(\frac{{\left[K^{+}\right]}_{apo}}{{\left[K^{+}\right]}_{cell}}\right)+V_{0})∙(1-trigger)\right)∙dt$$

### Simulation

For the numerical simulations, we set α = 15, *V*_*excited*_ = −60 mV, *V*_*rest*_ = −170 mV, [*K*^*+*^]_*cell*,*0*_ = 100 mM, [*K*^*+*^]_*apo*,*0*_ = 1 mM, ⇒ *V*_*0*_ = 55 mV. Other reasonable values do not change the results qualitatively. With a value of *a*_*K*_/*Vol*_*cell*_ = 1 mol/(l·V·s), the whole cell K^+^ current (without compensating anion current) across the membrane of a cell with a cytosol of 50 fl (10% of the total cell volume of 0.5 pl) under voltage clamp with *V*-*E*_*K*_ = 50 mV would be approximately 250 pA, which is well in the observed physiological range. Under normal conditions, the uptake rate might be smaller by a factor of approximately 100, i.e., β_normal_ = 0.01.

The system started at equilibrium in the resting state, i.e., *trigger* = 0. The pressure difference at the beginning is given by

$${\Delta P}_{0}=RT∙(c_{i,0}-c_{o,0})=RT∙\left(\frac{n_{i,0}}{{Vol}_{cell}}-\frac{n_{o,0}}{\alpha ∙{Vol}_{cell}}\right)=\frac{RT}{{Vol}_{cell}}∙\left(n_{i,0}-\frac{n_{o,0}}{\alpha }\right)=\frac{RT}{{Vol}_{cell}}∙{\Delta n}_{0},$$ (10)

which can be estimated to be approximately 5 bar. Here, *n*_*i*,0_ and *n*_*o*,0_ denote the osmotically active molar amount of substance within and outside the cell, respectively. Δ*n*_0_ is the difference *n*_*i*,0_ –*n*_*o*,0_/α. Upon stimulation of the trigger hair, the cell gets excited for approximately 1 second. In the simulation, this situation has been approximated by setting the membrane voltage for *t* = 1 s to *V*_*excited*_ and *trigger* = 1, which induced a K^+^/A^−^ efflux from the cell. The changes in the K^+^ concentrations and the pressure difference were calculated by iteratively solving the differential Eqs (5–9) with a time span of *dt* = 0.05 s.

## Supporting information

S1 Fig Trigger hair is unique among other tissues. (A) PCA of all replicates from all the studied tissues of *D*. *muscipula* (flower, root, petiole, trap, rim of the trap, glands, and trigger hairs) shows that the 3 replicates of each tissue strongly group together and that they exhibit a general low variance within each replicate group. The expression of all genes as rlog calculated by DEseq2 R package for each replicate was used. (B) Pearson correlation coefficient values of pairwise comparisons for each of the analyzed tissues shows that the trigger hair is highly different from all the other tissues, except for the trap (0.76). A Pearson correlation coefficient value close to 1 = high similarity (narrow ellipse and darker gray color) and close to 0 = high dissimilarity (wide ellipse and lighter gray color). The expression of all genes as mean FPKM values for each group was used. Underlying raw dataset is provided in S4 Data. FPKM, Fragments Per Kilobase of transcript per Million mapped reads; PCA, principal component analysis; rlog, regularized logarithm.
(TIF)

S2 Fig Phylogeny for the plant Shaker family including *D*. *muscipula* K^+^ channels. The phylogenetic tree displays speciation (S) or gene duplication (D) events for each branch node which are indicated by blue (S) or red dots (D). Next to the sequence labels, the placement and identity of the protein domains are indicated by colored boxes. The intervening sequence is shown as a solid line. On the right side, the trimmed sequence alignments are indicated by black boxes representing the aligned sequence, whereas the white regions with black dashed lines indicate gaps. The *D*. *muscipula* Shaker K^+^ channels are highlighted in yellow, and only KDM1 could be found in the K_in_-a subclade. Phylogenetic analysis was performed as described recently in [16].
(TIF)

S3 Fig Trigger hair–specific channels are highly expressed in the basal part. (A) *D*. *muscipula* trigger hair comprising 2 major parts: the tip, forming a lever structure which amplifies the signal, and the trigger hair base, comprising the indentation zone with the sensory cells. (B) qPCR relative expression of trigger hair–specific genes in the whole trigger hair, in the tip of the trigger hair, and in the base of the trigger hair. For the whole trigger hairs, 300–600 trigger hairs were needed for 1 replicate, while for the tip and the base parts, between 880 and 940 trigger hairs were needed to extract enough RNA for 1 replicate (*n* = 3; mean normalized to 10,000 actin ± SE). The full raw data are provided in S3 Data. qPCR, quantitative polymerase chain reaction; SE, standard error.
(TIF)

S4 Fig The trigger hair transportome. (A) Table showing transporters, channels, and pumps classified according to Aramemnon plant membrane protein database nomenclature that are specific to the trigger hair (Shannon entropy Q_gene|hair_-value < 3.9, hair expression, FPKM > 20). Out of a total of 313 genes, 22 were classified as part of the transportome under the mentioned thresholds. The “Nr.” column represents the number of members in each gene family/superfamily, the “Superfamily/Family” and “Family/Group” columns represent the Aramemnon classification nomenclature for plant membrane proteins, the “Gene Names” column represents *A*. *thaliana* homologs that were the “best hit” within the Mercator 3.6 annotation procedure, the “DmID” column represents the *D*. *muscipula* gene identifiers according to the reference genome, the “Hair FPKM” column represents the average expression level of each gene as FPKM in the trigger hair tissue, and the “Q_gene|hair_” column represents the specificity level according to the Shannon entropy method for tissues specificity where low values represent high specificity. (B) Doughnut chart showing the proportion of each major Superfamily/Family. FPKM, Fragments Per Kilobase of transcript per Million mapped reads.
(TIF)

S5 Fig *D*. *muscipula* GCs operate stomatal opening independently of KDM1. qPCR relative expression of GC marker genes in isolated GC from trap tissue and GC from petiole tissue compared to expression in the entire trap and in the entire petiole (*n* ≥ 7; mean normalized to 10,000 actin ± SE). *DmQUAC* and *DmSLAC1* were significantly enriched in the GCs of both trap (Mann–Whitney *p* = 0.004938 and 0.0106, respectively) and petiole (Mann–Whitney *p* = 0.0005784 and 0.0008053, respectively), whereas *DmSKOR* was enriched in the non-GC tissue (both entire trap Mann–Whitney *p* = 0.01519 and entire petiole *p* = 0.03322). KDM1 expression was not statistically significant in any of the GC when compared to non-GC tissues like entire traps (Mann–Whitney *p* = 0.4433) and entire petioles (Mann–Whitney *p =* 0.1743). The full raw data are provided in S3 Data. GC, guard cell; qPCR, quantitative polymerase chain reaction; SE, standard error.
(TIF)

S6 Fig KDM1 revealed typical electrophysiological features of the K_in_-a subclade. (A) With increasing external K^+^ concentration, the inward currents mediated by KDM1 also increased. The measured I_SS_ were normalized to −150 mV and 100 mM K^+^ at pH 4 (*n* = 4; mean ± SD). (B) Analyzing the relative open probability in 3 mM and 100 mM external K^+^ at a pH of 4 revealed that KDM1 exhibits no K^+^_ext_ dependent gating (n ≥ 7; mean ± SD). (C) The determination of the reversal potential by plotting the tail currents (I_tail_) against the applied K^+^ concentrations revealed a shift toward more negative membrane potentials with decreasing the external K^+^ concentration. The I_tail_ were normalized to −150 mV and 100 mM K^+^ at pH 4 (*n* = 4; mean ± SD). (D) Selectivity analyses of KDM1 expressing oocytes exhibited the highest current amplitude in the K^+^-containing buffer, whereas KDM1 was less permeable for the monovalent cations NH_4_^+^ and Rb^+^ and not conductive for Li^+^ and Na^+^ ions. The steady state currents (I_SS_) were normalized for each single cell to −150 mV and 30 mM KCl at pH 4 and plotted against the applied voltages (*n* = 7 for K^+^, NH_4_^+^, Rb^+^, Li^+^; *n* = 3 for Na^+^; mean ± SD). The full raw data are provided in S3 Data. SD, standard deviation.
(TIF)

S7 Fig KDM1 is pH sensitive. (A) Normalized currents of KDM1 expressing oocytes at indicated potentials in 100 mM KCl. I_SS_ were normalized to −150 mV and pH 4 to elucidate the altered pH sensitivity between the KDM1 WT and the H147S mutant (c.f. S8D Fig). Stepwise acidification from pH 7 to 4 increased the potassium currents through KDM1 (*n* = 20; mean ± SD). (B) The relative open probabilities of KDM1 expressing oocytes at the indicated H^+^ concentrations were plotted against the applied test voltage. Note the prominent positive shift of the half-maximal activation potential (V_1/2_) with increasing acidification. The data points were fitted with a Boltzmann function (solid lines; *n* = 21; mean ± SD). The full raw data are provided in S3 Data. SD, standard deviation.
(TIF)

S8 Fig Sequence and structure comparison of *D*. *muscipula* KDM1 with orthologous plant channels: Focused view of a sequence alignment of plant KAT1 orthologues channel proteins showing the S3 helix and the S5-pore helix region. (A) Plant KAT1-like voltage-dependent K^+^ channels exhibit a highly conserved proline residue within the S3 helix (red arrow), which serves as a helix breaking point and splits S3 into a longer helix-a and a shorter helix-b (see also (B)). H147, unique for *D*. *muscipula* KDM1 is located in S3 helix-b toward the extracellular side of the channel. A previously identified pH-sensing H160 in the potato guard cell KST1 channel locates in the S3 and S4 linker regions. H223 and H237, shown not to be involved in pH-regulated, voltage-dependent gating of KDM1 locate in the linker region connecting helix S5 and the pore helix (A). (B) Structural model of the KDM1 tetramer (green), based on the *A*. *thaliana* KAT1 structure (cyan) [20] showing the position of the histidine residues investigated in this study. The H147 containing S3 helix is depicted in yellow (KAT1) or orange (KDM1). For the sake of clarity, histidine residues are shown for 2 subunits only. (C) Currents mediated by the KDM1 mutant H147S were normalized to −150 mV at pH 4 and plotted against the different test pulses. The I/V curve of H147S mutant at different pH values and a constant external K^+^ concentration indicated that K^+^ influx in the mutant is less affected by changes in the external proton concentration compared to WT KDM1 (c.f. S7A Fig) (*n* = 11; mean ± SD). Comparing unnormalized I_SS_ of the WT channel and the H147S mutant, at −150 mV, 100 mM KCl, and pH 4, WT KDM1 mediated −15 μA ± 1.7 (mean ± SE; *n* = 36), whereas the K^+^ current of the mutant was reduced by a factor of 0.67 to −10 μA ±2.1 (mean ± SE; *n* = 20)). (D) Relative open probability of KDM1 H147S expressing oocytes at the indicated H^+^ concentrations plotted against the applied voltages. The pH-dependent shift of V_1/2_ with only −31 mV in the H147S mutant was about 50% compared to the WT (c.f. S7B Fig). The data points were fitted with a Boltzmann function (solid lines; *n* = 14; mean ± SD). The full raw data are provided in S3 Data. SD, standard deviation; SE, standard error; WT, wild-type.
(TIF)

S9 Fig The impact of K^+^ channel blockers on the KDM1 activity. (A) to (D) show macroscopic currents of a representative cell in 30 mM and 100 mM K^+^ with and without 30 mM Cs^+^. At hyperpolarized voltages, Cs^+^ application led to no potassium influx, and the tail currents revealed the voltage-dependent block. (E) The relative open probability was plotted against the applied voltages under the indicated conditions. The half-activation potential V_1/2_ was not much affected by Cs^+^ application (*n* ≥ 4; mean ± SD). (F) The unnormalized I_SS_ in 30 mM and 100 mM K^+^, with and without 30 mM Cs^+^, displays comparable current characteristics as the normalized I_SS_-V curves in Fig 4A (*n* = 17; mean ± SD). (G) KDM1-mediated tail currents (normalized to −150 mV and 30 mM K^+^) at 100 mM or 30 mM external K^+^ with or without 30 mM Cs^+^. The I_tail_ shows inward-directed potassium currents, which decreased in a voltage-dependent manner under Cs^+^ application. This reduction in the tail currents is more pronounced when 30 mM K^+^ together with 30 mM Cs^+^ was applied (*n* = 17; mean ± SD). (H) The same current characteristics could be observed by plotting the unnormalized I_tail_ against the test voltages (*n* = 17; mean ± SD). (I) Steady state currents at −150 mV in the presence of the indicated blocker (30 mM each) were normalized to −150 mV and 30 mM K^+^. The application of TEA^+^ and Ba^2+^ reduced the current by about 60%, whereas Cs^+^ blocked the channel activity by about 90% (*n* ≥ 6; mean ± SD). (J) KDM1-mediated tail currents were normalized to −150 mV and 30 mM K^+^ at pH 4. The currents were recorded in 30 mM K^+^, with or without 30 mM Cs^+^, at pH 4 and 6. Due to the negatively shifted open probability at pH 6, no K^+^ influx could be detected in the presence of Cs^+^ (*n* = 7; mean ± SD). The full raw data are provided in S3 Data. SD, standard deviation.
(TIF)

S10 Fig Cs^+^ blocks AP generation in trigger hairs. A *D*. *muscipula* trap was fixed in an open position using adhesive tape and cut from the outer rim to the midrib close to a trigger hair (left). Two surface potential electrodes (red and black) were placed in close proximity to 2 trigger hairs (TH1 and TH2), and a droplet of 30 mM Cs^+^ was applied at the cut edge close to TH1. Mechanical stimulation of TH1 at time point 15 seconds resulted in action potential (AP) generation detected at both electrodes. After 570 seconds of Cs^+^ treatment, no AP was elicited in TH1. Stimulation of TH2 at 675 seconds resulted in AP generation, which was also detectable at both sites indicating that Cs^+^ just toxified TH1 rather than the nearby trap tissue. Wounding in tissue close to TH1 also resulted in AP generation which was detectable at both sites (1,045 seconds). AP, action potential.
(TIF)

S11 Fig Cs^+^ blocks AP generation in trigger hairs. An intact trigger hair (serving as “AP receiver”) was impaled in the indentation zone of the base and the membrane potential (AP) was recorded. Alongside both other trigger hairs (TH1 and TH2—serving as independent “AP actuators”), the trap was cut to ensure the toxification of TH1 and TH2 approximately at the same time after applying 30 mM Cs^+^. Three APs were triggered in control conditions by bending the “actuator” TH1 prior to adding Cs^+^ to the solution. Further APs were triggered manually at TH1 every 5 minutes until mechanosensory cells failed to elicit an AP, followed by the observation that the bending of the untreated “AP actuator.” TH2 also no longer evoked an AP. The electrical network of the trap tissue still responded with APs triggered by heat or wounding. This set of experiments was repeated 3 times, and a representative trace is shown here. AP, action potential.
(TIF)

S1 Table qPCR conditions. qPCR conditions for expression analysis together with forward and reverse primers used for the expression analysis of each investigated gene. qPCR, quantitative polymerase chain reaction.
(XLSX)

S2 Table FastQC output for trigger hair replicates. Sequencing Quality Check data (FastQC output; see Materials and methods section) together with the number of reads for each of the paired-end of the trigger hair replicates are shown.
(XLSX)

S1 Movie Cs^+^ blocks AP generation in trigger hairs. Trigger hairs were immersed in 1.5% low melting agarose 30 mM Cs^+^ for 100 minutes. No AP was generated after bending Cs^+^-treated sensory hairs resulting in no trap closure. AP, action potential.
(MP4)

S2 Movie One unblocked trigger hair is sufficient for trap closure. Cs^+^ treatment applied locally at the trigger hair level blocks trigger hair touch-induced trap closure, while the electrical trap network interconnecting the trigger hairs remains fully functional. Five trigger hairs within 1 trap were covered with 1.5% low melting agarose 30 mM Cs^+^, and all became electrically silent. Mechanostimulation of the untreated sixth trigger hair, however, elicited an AP sufficient to provoke trap closure. AP, action potential.
(MP4)

S3 Movie Cs^+^ does not impair the electrical trap network. Cs^+^ treatment of trigger hairs does not impair trap closure provoked by exerting a force on the trap lobe. Covering all trigger hairs with 1.5% low melting agarose 30 mM Cs^+^ completely suppressed AP firing via trigger hair stimulation, resulting in no trap closure, while using a forceps to squeeze the trap lobes twice still elicited APs leading to trap closure. AP, action potential.
(MP4)

S1 Data Underlying data used for generating Fig 1. S1A Data contains the raw output of DEseq2 R package used for differential expression analysis for each of the 6 pairwise comparisons: hair (trigger hair) vs. flower, hair vs. glands, hair vs. petiole, hair vs. petiole, hair vs. root, and hair vs. trap. The last column indicates if a gene is part of subset02 of the intersection analysis (hair vs. all other tissues) (see Fig 1). For column names description, see DEseq2 manual. S1B Data contains the raw output of Ontologizer used for GO term enrichment analysis for each subset of the intersection plot. Only GO terms with a corrected BH adjusted *p*-value < 0.05 are listed. BH, Benjamini–Hochberg; GO, gene ontology.
(XLSX)

S2 Data Underlying raw data used for generating Fig 2. Expression values (FPKM) of all the 21,135 genes in root, flower, petiole, trap, rim, glands, and trigger hair, as well as pairwise differential expression log_2_FC values of each comparison (trigger hair vs. each of all the other tissues) that pass a threshold of BH *p*-adjusted value < 0.001 are shown. The Shannon entropy calculated H_gene_-value, and Q_gene|hair_-values for the trigger hair is also shown. All the genes are annotated according to the Mercator 3.6 annotation tool including *A*. *thaliana* homologs and gene names together with their description. All the transcripts have been blasted against the Aramemnon plant membrane protein database; therefore, transportome members have been annotated accordingly. Bona fide trigger hair–specific genes identified by a combination of both methods (Shannon entropy together with the differential expression analysis—see Materials and methods section) are marked in red and ordered by Q_gene|hair_-value. An additional FPKM > 20 filter for the trigger hair expression level shows only the highly expressed genes that are also trigger hair specific. BH, Benjamini–Hochberg; FPKM, Fragments Per Kilobase of transcript per Million mapped reads; NA, not assigned.
(XLSX)

S3 Data Underlying full raw data and statistical analysis used for generating Figs 3D and 3E, 4A and 4D and 5D and S3B, S5, S6A–S6D, S7A and S7B, S8C and S8D, and S9E–S9J Figs. (XLSX)

S4 Data Underlying raw data used for generating S1 Fig. For PCA analysis (S1A Fig), the expression level of all genes as rlog calculated by DEseq2 R package for each replicate was used. For correlation plot (S1B Fig), the expression of all genes as mean FPKM values was used. FPKM, Fragments Per Kilobase of transcript per Million mapped reads; PCA, principal component analysis; rlog, regularized logarithm.
(XLSX)

## Abbreviations

ABC: ATP-binding cassette
AP: action potential
ATPase: adenosine triphosphatase
BH: Benjamini–Hochberg
CaCA: Ca^2+^/cation antiporters
cryo-EM: cryogenic electron microscopy
DEG: differentially expressed gene
ER: endoplasmic reticula
FELASA: Federation of European Laboratory Animal Science Associations
FPKM: Fragments Per Kilobase of transcript per Million mapped reads
GLR: glutamate receptor‐like
GO: gene ontology
KUP: K^+^ uptake transporter
MSL: Mechanosensitive Channel of Small Conductance-Like
PCA: principal component analysis
PD: pore domain
PM: plasma membrane
PVC: polyvinyl chloride
qPCR: quantitative polymerase chain reaction
RNA-seq: RNA sequencing
RQI: RNA quality indicator
SRA: Sequence Read Archive
TEVC: Two-Electrode-Voltage-Clamp
TierSchVersV: Tierschutz-Versuchstierverordnung
VSD: voltage-sensing domain
WT: wild-type
YFP: yellow fluorescent protein
