# Supplementary figures and images for: The Venus flytrap trigger hair–specific potassium channel KDM1 can reestablish the K+ gradient required for hapto-electric signaling
Source: PLoS Biol. 2020 Dec 9;18(12):e3000964. doi: 10.1371/journal.pbio.3000964 (PMC7725304; doi:10.1371/journal.pbio.3000964)

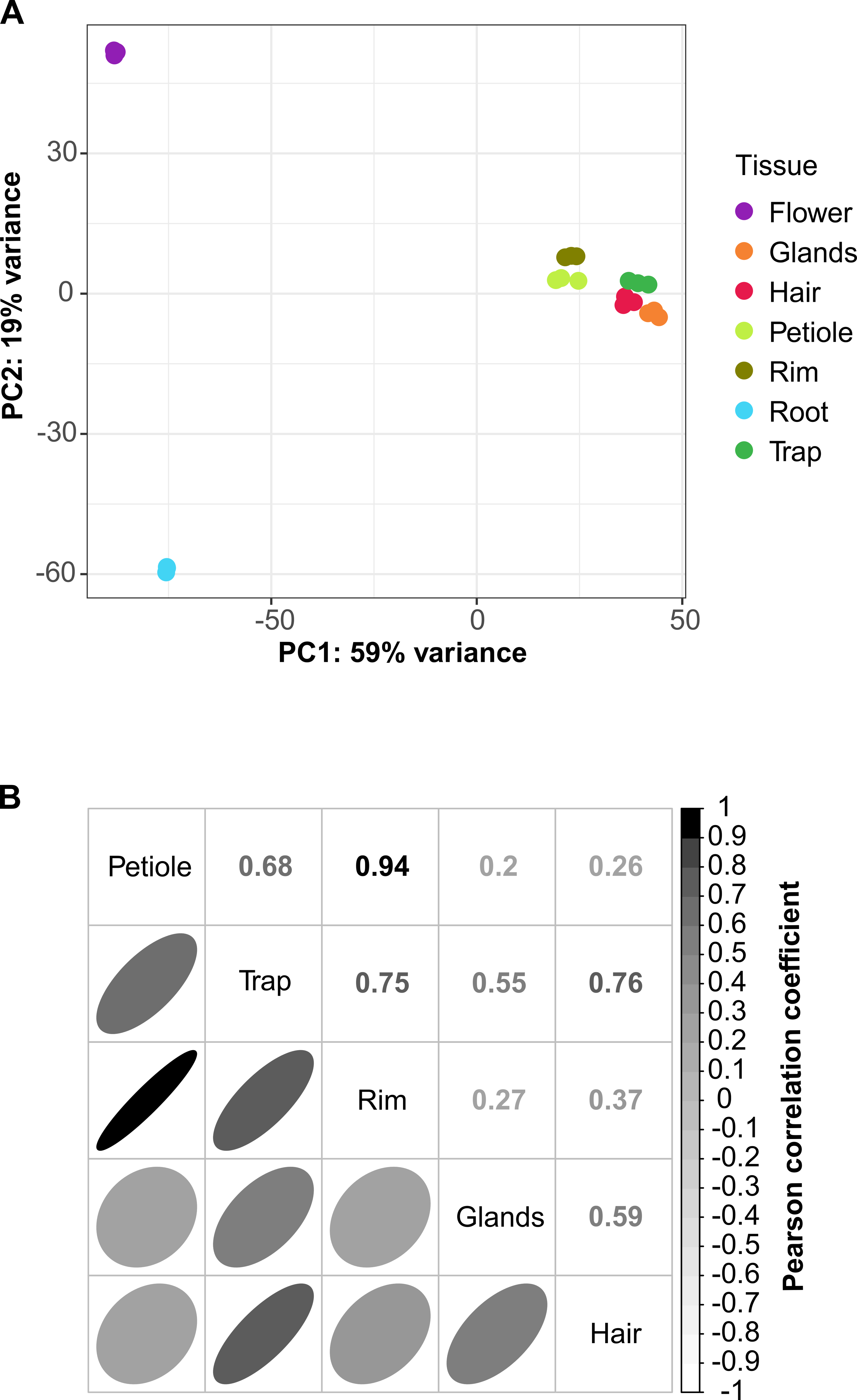

Supplement: S1 Fig — (A) PCA of all replicates from all the studied tissues of D. muscipula (flower, root, petiole, trap, rim of the trap, glands, and trigger hairs) shows that the 3 replicates of each tissue strongly group together and that they exhibit a general low variance within each replicate group. The expression of all genes as rlog calculated by DEseq2 R package for each replicate was used. (B) Pearson correlation coefficient values of pairwise comparisons for each of the analyzed tissues shows that the trigger hair is highly different from all the other tissues, except for the trap (0.76). A Pearson correlation coefficient value close to 1 = high similarity (narrow ellipse and darker gray color) and close to 0 = high dissimilarity (wide ellipse and lighter gray color). The expression of all genes as mean FPKM values for each group was used. Underlying raw dataset is provided in S4 Data. FPKM, Fragments Per Kilobase of transcript per Million mapped reads; PCA, principal component analysis; rlog, regularized logarithm. (TIF) [file pbio.3000964.s001.tif]

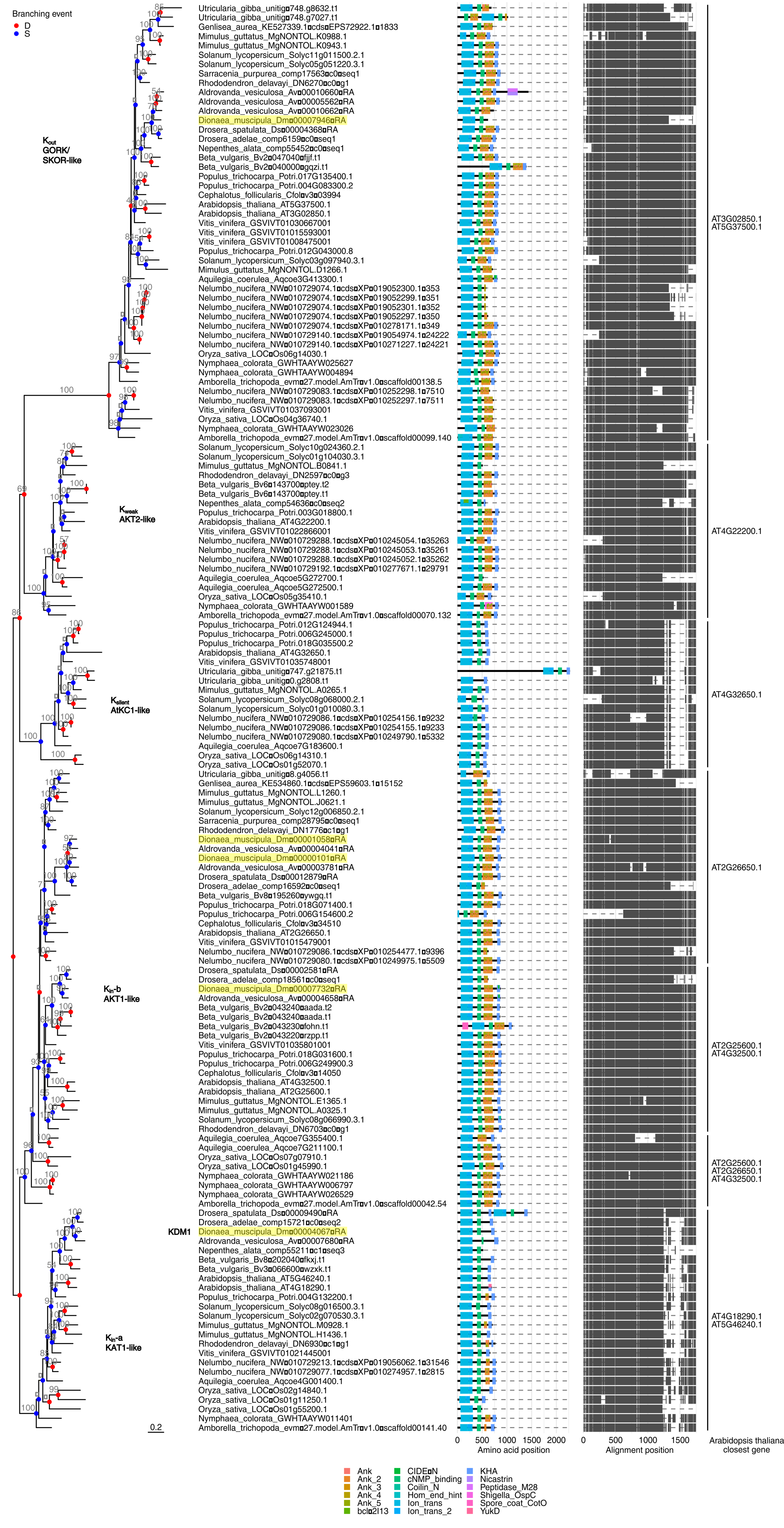

Supplement: S2 Fig — The phylogenetic tree displays speciation (S) or gene duplication (D) events for each branch node which are indicated by blue (S) or red dots (D). Next to the sequence labels, the placement and identity of the protein domains are indicated by colored boxes. The intervening sequence is shown as a solid line. On the right side, the trimmed sequence alignments are indicated by black boxes representing the aligned sequence, whereas the white regions with black dashed lines indicate gaps. The D. muscipula Shaker K+ channels are highlighted in yellow, and only KDM1 could be found in the Kin-a subclade. Phylogenetic analysis was performed as described recently in [16]. (TIF) [file pbio.3000964.s002.tif]

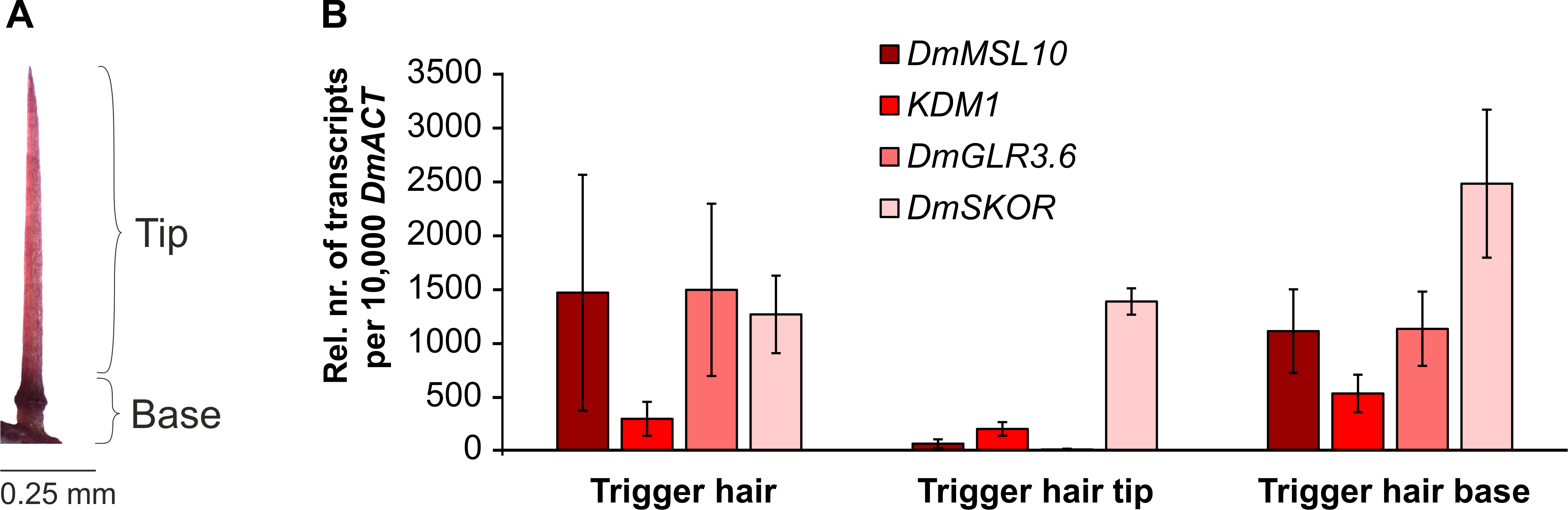

Supplement: S3 Fig — (A) D. muscipula trigger hair comprising 2 major parts: the tip, forming a lever structure which amplifies the signal, and the trigger hair base, comprising the indentation zone with the sensory cells. (B) qPCR relative expression of trigger hair–specific genes in the whole trigger hair, in the tip of the trigger hair, and in the base of the trigger hair. For the whole trigger hairs, 300–600 trigger hairs were needed for 1 replicate, while for the tip and the base parts, between 880 and 940 trigger hairs were needed to extract enough RNA for 1 replicate (n = 3; mean normalized to 10,000 actin ± SE). The full raw data are provided in S3 Data. qPCR, quantitative polymerase chain reaction; SE, standard error. (TIF) [file pbio.3000964.s003.tif]

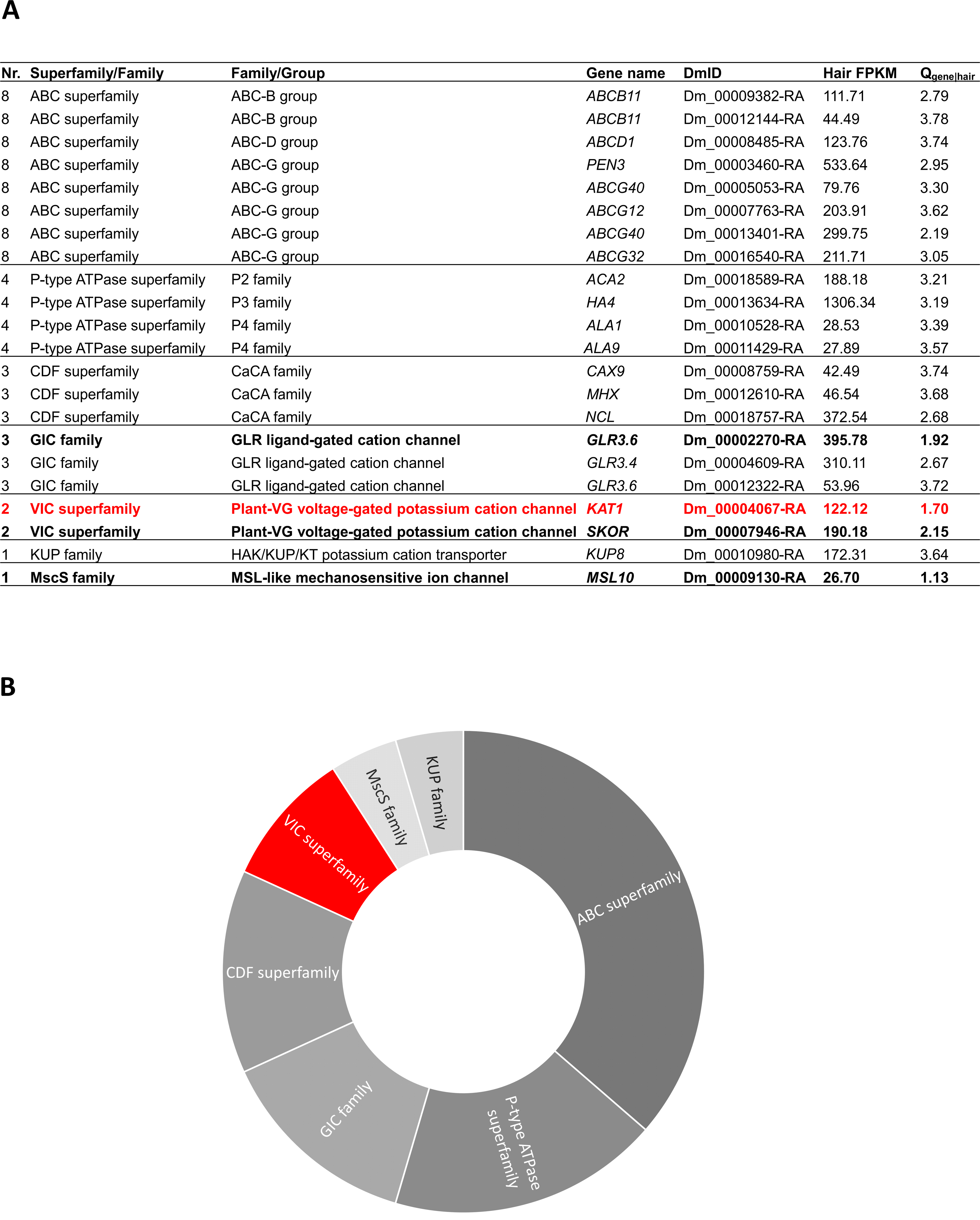

Supplement: S4 Fig — (A) Table showing transporters, channels, and pumps classified according to Aramemnon plant membrane protein database nomenclature that are specific to the trigger hair (Shannon entropy Qgene|hair-value < 3.9, hair expression, FPKM > 20). Out of a total of 313 genes, 22 were classified as part of the transportome under the mentioned thresholds. The “Nr.” column represents the number of members in each gene family/superfamily, the “Superfamily/Family” and “Family/Group” columns represent the Aramemnon classification nomenclature for plant membrane proteins, the “Gene Names” column represents A. thaliana homologs that were the “best hit” within the Mercator 3.6 annotation procedure, the “DmID” column represents the D. muscipula gene identifiers according to the reference genome, the “Hair FPKM” column represents the average expression level of each gene as FPKM in the trigger hair tissue, and the “Qgene|hair” column represents the specificity level according to the Shannon entropy method for tissues specificity where low values represent high specificity. (B) Doughnut chart showing the proportion of each major Superfamily/Family. FPKM, Fragments Per Kilobase of transcript per Million mapped reads. (TIF) [file pbio.3000964.s004.tif]

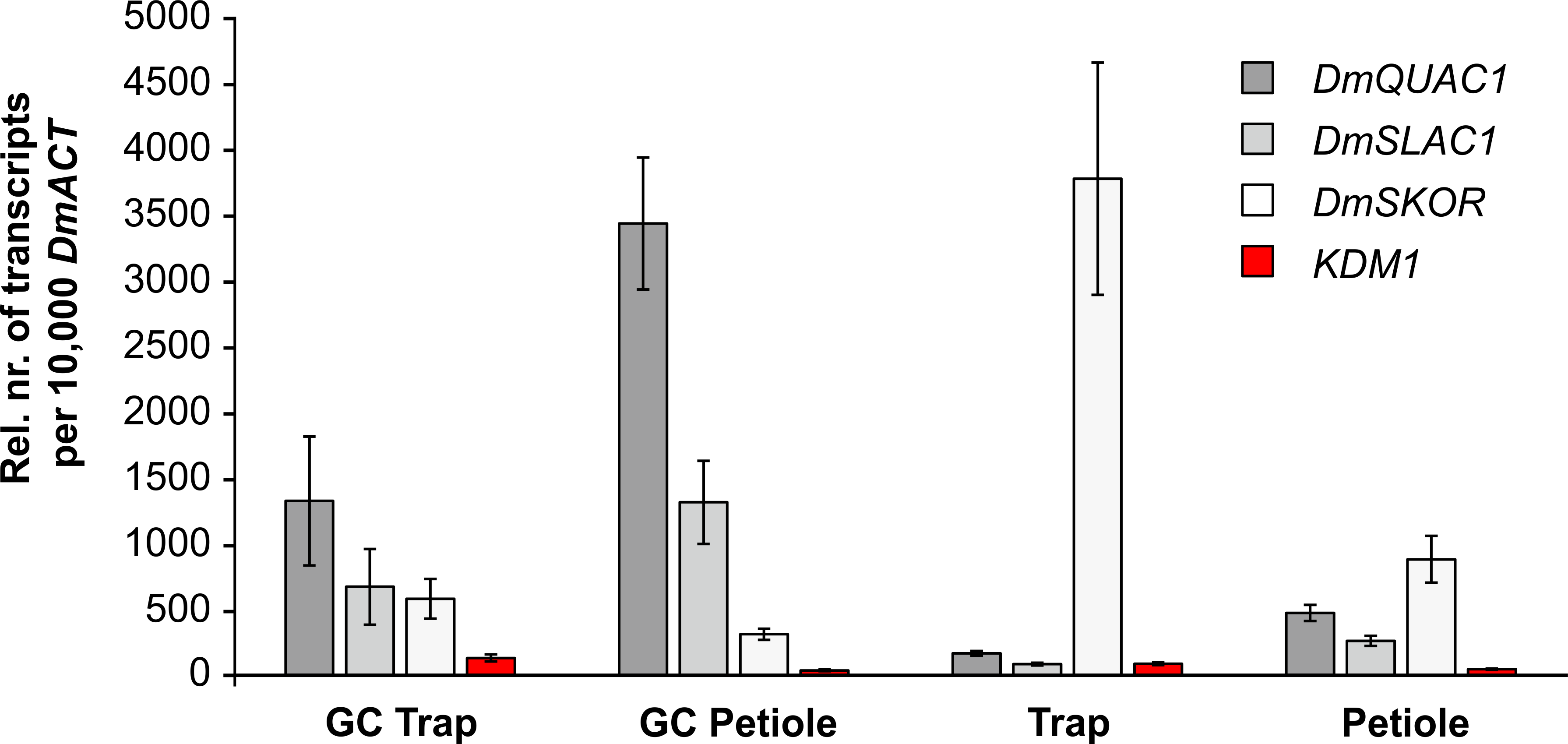

Supplement: S5 Fig — qPCR relative expression of GC marker genes in isolated GC from trap tissue and GC from petiole tissue compared to expression in the entire trap and in the entire petiole (n ≥ 7; mean normalized to 10,000 actin ± SE). DmQUAC and DmSLAC1 were significantly enriched in the GCs of both trap (Mann–Whitney p = 0.004938 and 0.0106, respectively) and petiole (Mann–Whitney p = 0.0005784 and 0.0008053, respectively), whereas DmSKOR was enriched in the non-GC tissue (both entire trap Mann–Whitney p = 0.01519 and entire petiole p = 0.03322). KDM1 expression was not statistically significant in any of the GC when compared to non-GC tissues like entire traps (Mann–Whitney p = 0.4433) and entire petioles (Mann–Whitney p = 0.1743). The full raw data are provided in S3 Data. GC, guard cell; qPCR, quantitative polymerase chain reaction; SE, standard error. (TIF) [file pbio.3000964.s005.tif]

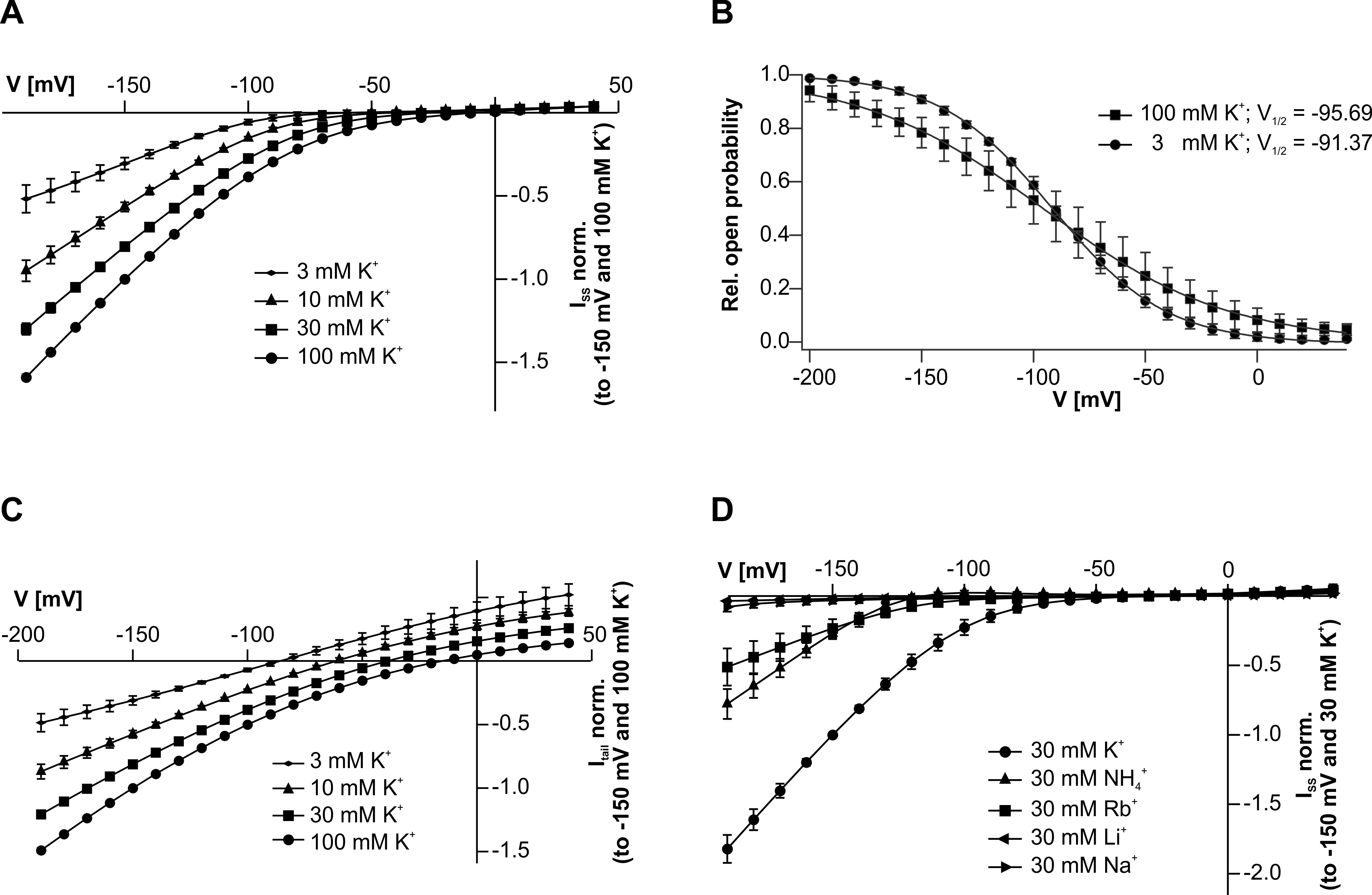

Supplement: S6 Fig — (A) With increasing external K+ concentration, the inward currents mediated by KDM1 also increased. The measured ISS were normalized to −150 mV and 100 mM K+ at pH 4 (n = 4; mean ± SD). (B) Analyzing the relative open probability in 3 mM and 100 mM external K+ at a pH of 4 revealed that KDM1 exhibits no K+ext dependent gating (n ≥ 7; mean ± SD). (C) The determination of the reversal potential by plotting the tail currents (Itail) against the applied K+ concentrations revealed a shift toward more negative membrane potentials with decreasing the external K+ concentration. The Itail were normalized to −150 mV and 100 mM K+ at pH 4 (n = 4; mean ± SD). (D) Selectivity analyses of KDM1 expressing oocytes exhibited the highest current amplitude in the K+-containing buffer, whereas KDM1 was less permeable for the monovalent cations NH4+ and Rb+ and not conductive for Li+ and Na+ ions. The steady state currents (ISS) were normalized for each single cell to −150 mV and 30 mM KCl at pH 4 and plotted against the applied voltages (n = 7 for K+, NH4+, Rb+, Li+; n = 3 for Na+; mean ± SD). The full raw data are provided in S3 Data. SD, standard deviation. (TIF) [file pbio.3000964.s006.tif]

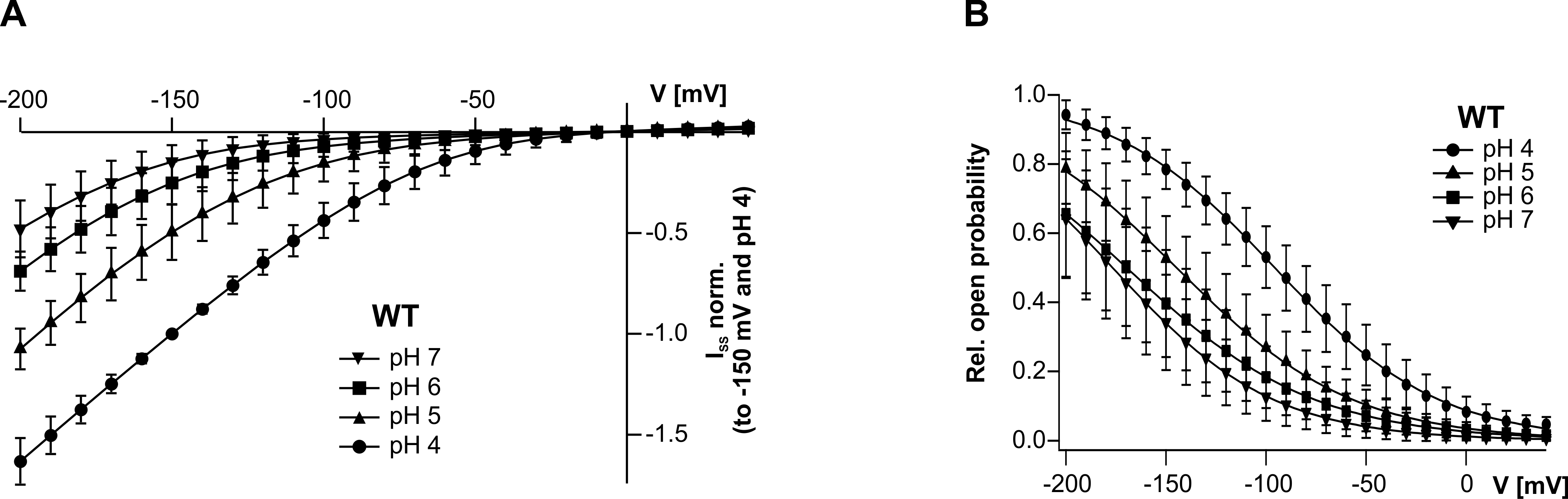

Supplement: S7 Fig — (A) Normalized currents of KDM1 expressing oocytes at indicated potentials in 100 mM KCl. ISS were normalized to −150 mV and pH 4 to elucidate the altered pH sensitivity between the KDM1 WT and the H147S mutant (c.f. S8D Fig). Stepwise acidification from pH 7 to 4 increased the potassium currents through KDM1 (n = 20; mean ± SD). (B) The relative open probabilities of KDM1 expressing oocytes at the indicated H+ concentrations were plotted against the applied test voltage. Note the prominent positive shift of the half-maximal activation potential (V1/2) with increasing acidification. The data points were fitted with a Boltzmann function (solid lines; n = 21; mean ± SD). The full raw data are provided in S3 Data. SD, standard deviation. (TIF) [file pbio.3000964.s007.tif]

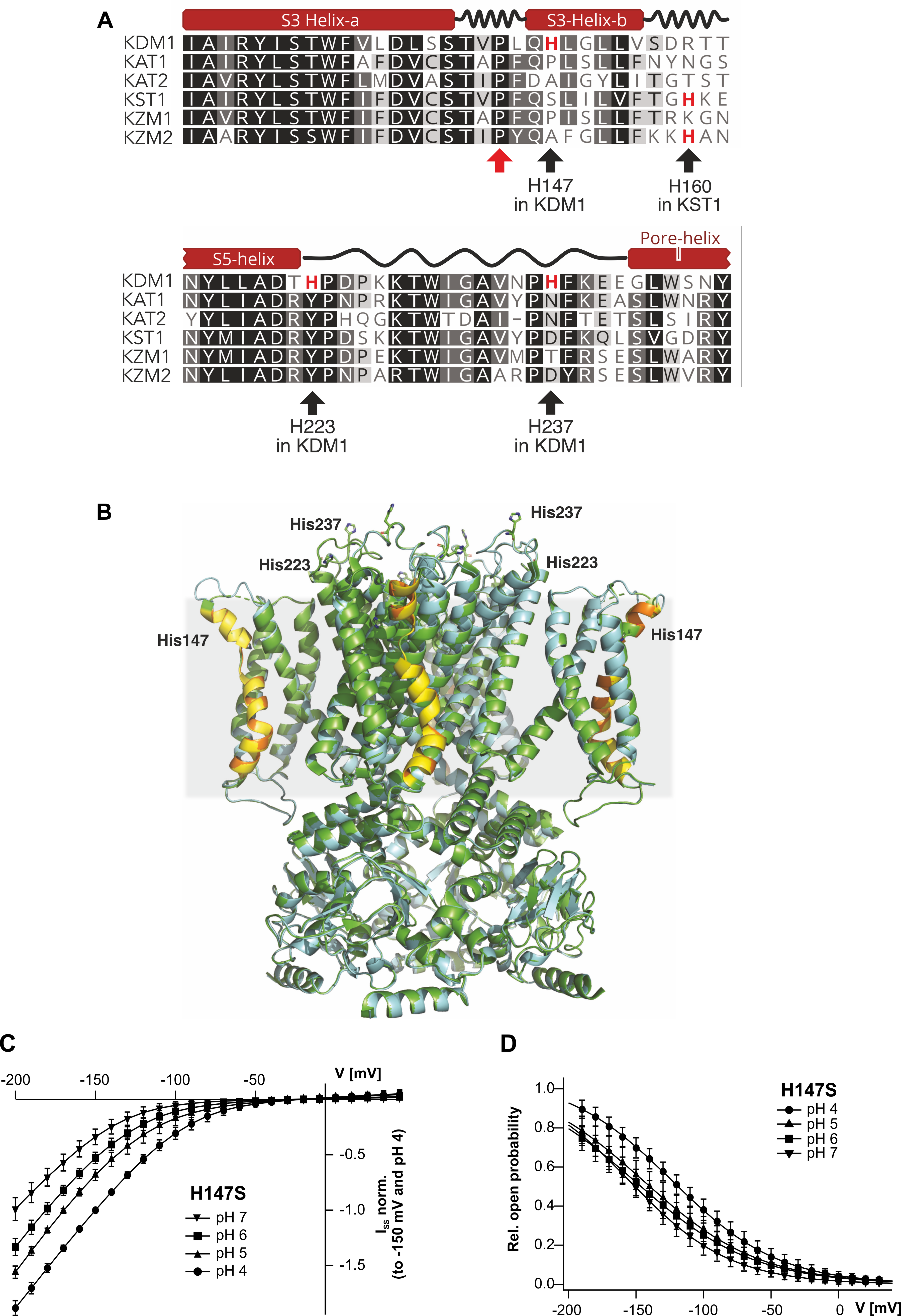

Supplement: S8 Fig — (A) Plant KAT1-like voltage-dependent K+ channels exhibit a highly conserved proline residue within the S3 helix (red arrow), which serves as a helix breaking point and splits S3 into a longer helix-a and a shorter helix-b (see also (B)). H147, unique for D. muscipula KDM1 is located in S3 helix-b toward the extracellular side of the channel. A previously identified pH-sensing H160 in the potato guard cell KST1 channel locates in the S3 and S4 linker regions. H223 and H237, shown not to be involved in pH-regulated, voltage-dependent gating of KDM1 locate in the linker region connecting helix S5 and the pore helix (A). (B) Structural model of the KDM1 tetramer (green), based on the A. thaliana KAT1 structure (cyan) [20] showing the position of the histidine residues investigated in this study. The H147 containing S3 helix is depicted in yellow (KAT1) or orange (KDM1). For the sake of clarity, histidine residues are shown for 2 subunits only. (C) Currents mediated by the KDM1 mutant H147S were normalized to −150 mV at pH 4 and plotted against the different test pulses. The I/V curve of H147S mutant at different pH values and a constant external K+ concentration indicated that K+ influx in the mutant is less affected by changes in the external proton concentration compared to WT KDM1 (c.f. S7A Fig) (n = 11; mean ± SD). Comparing unnormalized ISS of the WT channel and the H147S mutant, at −150 mV, 100 mM KCl, and pH 4, WT KDM1 mediated −15 μA ± 1.7 (mean ± SE; n = 36), whereas the K+ current of the mutant was reduced by a factor of 0.67 to −10 μA ±2.1 (mean ± SE; n = 20)). (D) Relative open probability of KDM1 H147S expressing oocytes at the indicated H+ concentrations plotted against the applied voltages. The pH-dependent shift of V1/2 with only −31 mV in the H147S mutant was about 50% compared to the WT (c.f. S7B Fig). The data points were fitted with a Boltzmann function (solid lines; n = 14; mean ± SD). The full raw data are provided in S3 Data. SD, standard deviati [file pbio.3000964.s008.tif]

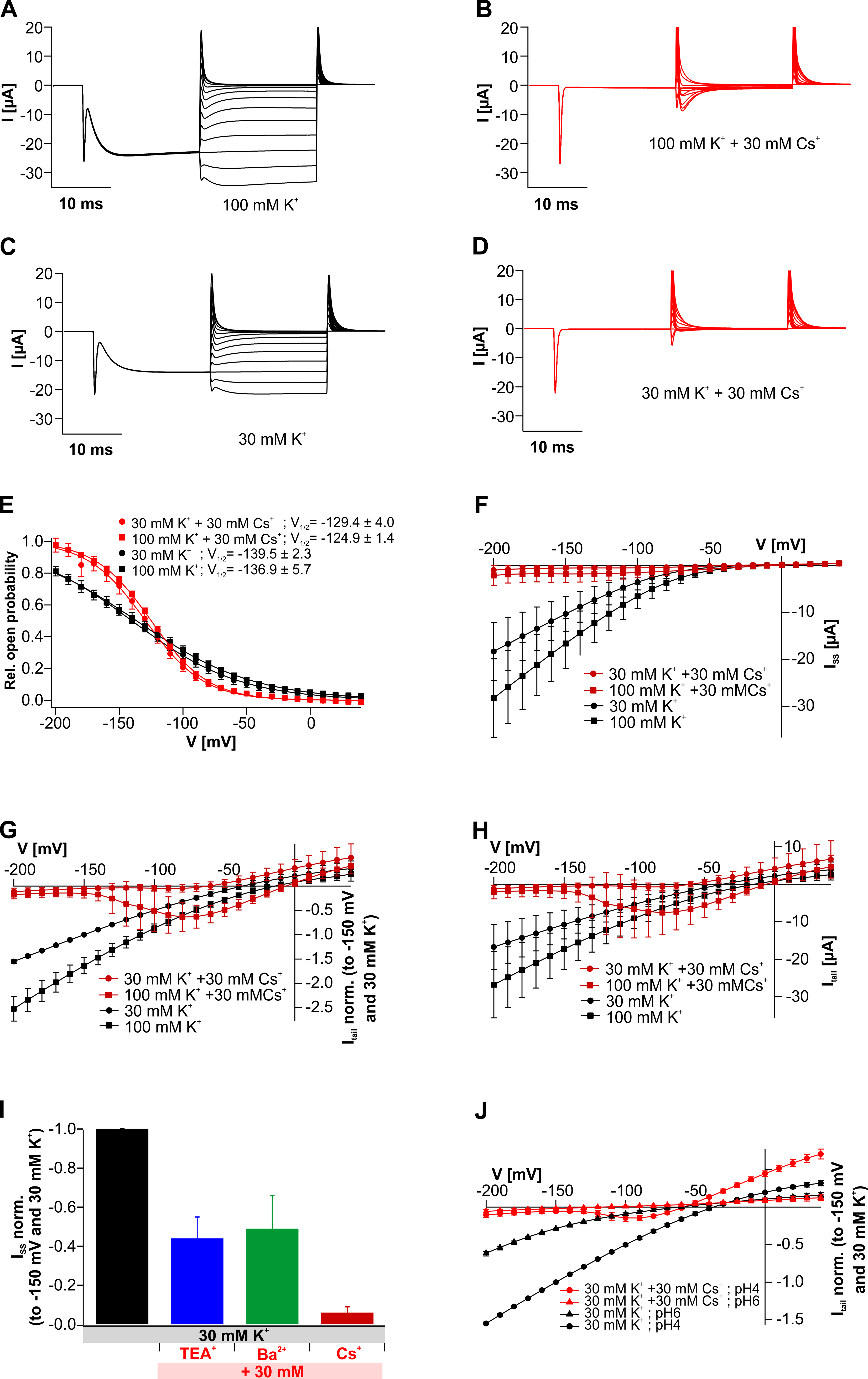

Supplement: S9 Fig — (A) to (D) show macroscopic currents of a representative cell in 30 mM and 100 mM K+ with and without 30 mM Cs+. At hyperpolarized voltages, Cs+ application led to no potassium influx, and the tail currents revealed the voltage-dependent block. (E) The relative open probability was plotted against the applied voltages under the indicated conditions. The half-activation potential V1/2 was not much affected by Cs+ application (n ≥ 4; mean ± SD). (F) The unnormalized ISS in 30 mM and 100 mM K+, with and without 30 mM Cs+, displays comparable current characteristics as the normalized ISS-V curves in Fig 4A (n = 17; mean ± SD). (G) KDM1-mediated tail currents (normalized to −150 mV and 30 mM K+) at 100 mM or 30 mM external K+ with or without 30 mM Cs+. The Itail shows inward-directed potassium currents, which decreased in a voltage-dependent manner under Cs+ application. This reduction in the tail currents is more pronounced when 30 mM K+ together with 30 mM Cs+ was applied (n = 17; mean ± SD). (H) The same current characteristics could be observed by plotting the unnormalized Itail against the test voltages (n = 17; mean ± SD). (I) Steady state currents at −150 mV in the presence of the indicated blocker (30 mM each) were normalized to −150 mV and 30 mM K+. The application of TEA+ and Ba2+ reduced the current by about 60%, whereas Cs+ blocked the channel activity by about 90% (n ≥ 6; mean ± SD). (J) KDM1-mediated tail currents were normalized to −150 mV and 30 mM K+ at pH 4. The currents were recorded in 30 mM K+, with or without 30 mM Cs+, at pH 4 and 6. Due to the negatively shifted open probability at pH 6, no K+ influx could be detected in the presence of Cs+ (n = 7; mean ± SD). The full raw data are provided in S3 Data. SD, standard deviation. (TIF) [file pbio.3000964.s009.tif]

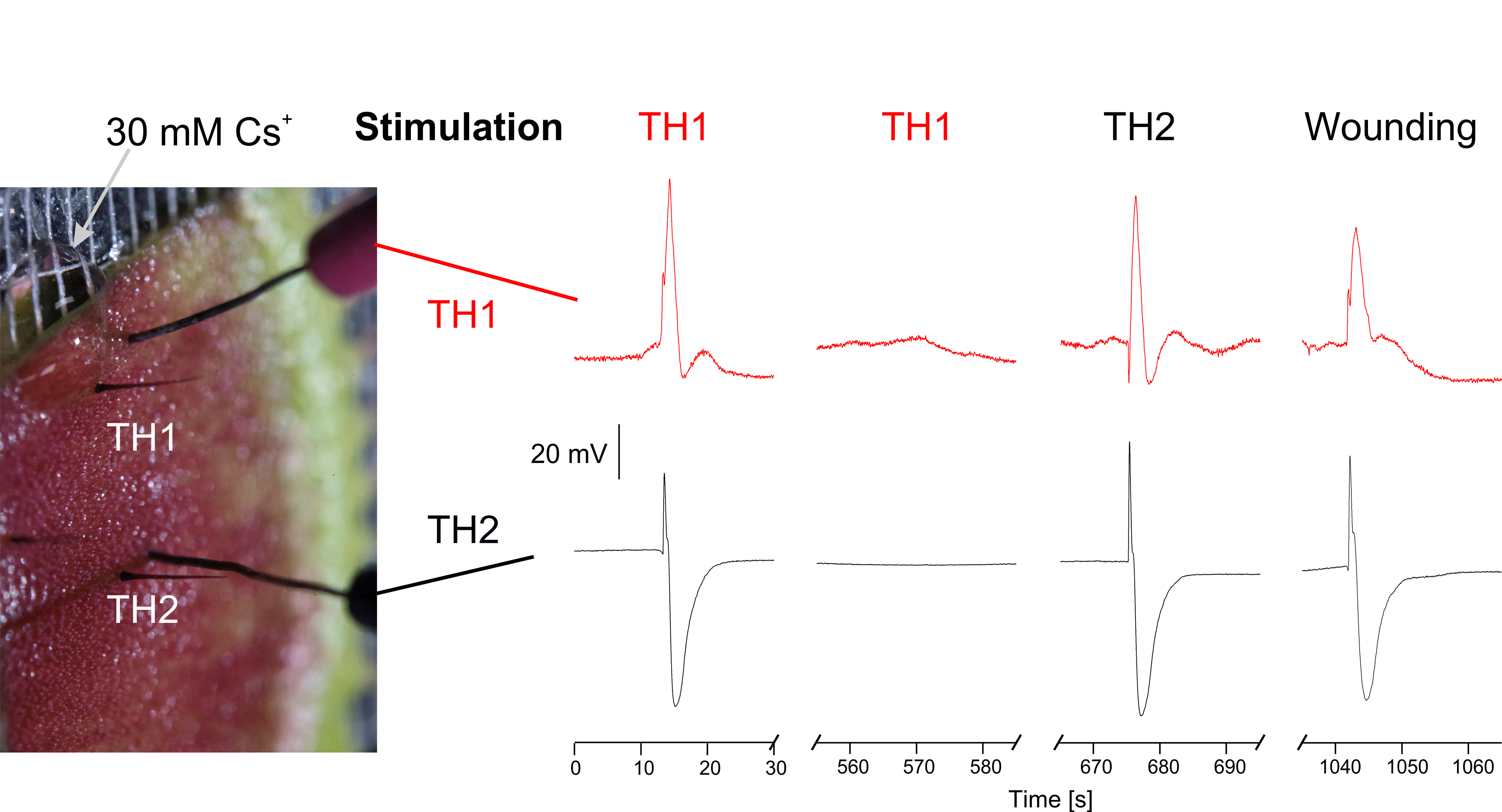

Supplement: S10 Fig — A D. muscipula trap was fixed in an open position using adhesive tape and cut from the outer rim to the midrib close to a trigger hair (left). Two surface potential electrodes (red and black) were placed in close proximity to 2 trigger hairs (TH1 and TH2), and a droplet of 30 mM Cs+ was applied at the cut edge close to TH1. Mechanical stimulation of TH1 at time point 15 seconds resulted in action potential (AP) generation detected at both electrodes. After 570 seconds of Cs+ treatment, no AP was elicited in TH1. Stimulation of TH2 at 675 seconds resulted in AP generation, which was also detectable at both sites indicating that Cs+ just toxified TH1 rather than the nearby trap tissue. Wounding in tissue close to TH1 also resulted in AP generation which was detectable at both sites (1,045 seconds). AP, action potential. (TIF) [file pbio.3000964.s010.tif]

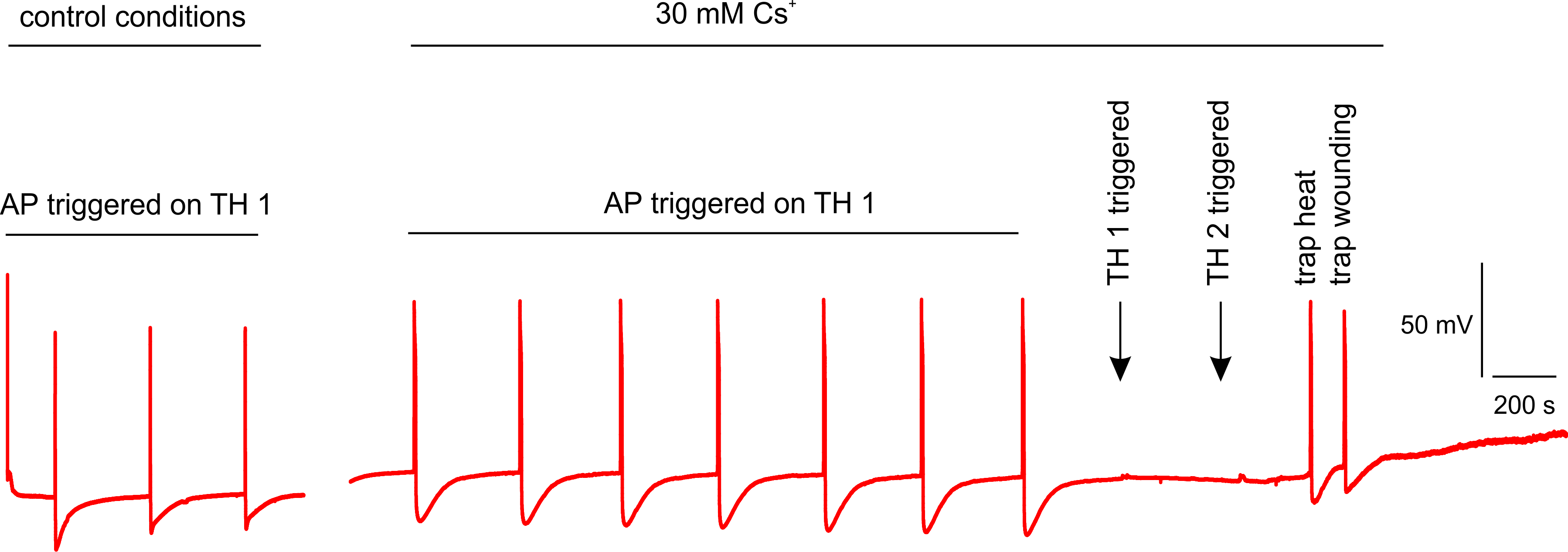

Supplement: S11 Fig — An intact trigger hair (serving as “AP receiver”) was impaled in the indentation zone of the base and the membrane potential (AP) was recorded. Alongside both other trigger hairs (TH1 and TH2—serving as independent “AP actuators”), the trap was cut to ensure the toxification of TH1 and TH2 approximately at the same time after applying 30 mM Cs+. Three APs were triggered in control conditions by bending the “actuator” TH1 prior to adding Cs+ to the solution. Further APs were triggered manually at TH1 every 5 minutes until mechanosensory cells failed to elicit an AP, followed by the observation that the bending of the untreated “AP actuator.” TH2 also no longer evoked an AP. The electrical network of the trap tissue still responded with APs triggered by heat or wounding. This set of experiments was repeated 3 times, and a representative trace is shown here. AP, action potential. (TIF) [file pbio.3000964.s011.tif]
